# Elovanoid-N34 modulates TXNRD1 key in protection against oxidative stress-related diseases

**DOI:** 10.1038/s41419-023-06334-6

**Published:** 2023-12-13

**Authors:** Jorgelina M. Calandria, Surjyadipta Bhattacharjee, Sayantani Kala-Bhattacharjee, Pranab K. Mukherjee, Yuehan Feng, Jakob Vowinckel, Tobias Treiber, Nicolas G. Bazan

**Affiliations:** 1 Neuroscience Center of Excellence, School of Medicine, Louisiana State University Health New Orleans, New Orleans, LA USA; 2 Biognosys AG, Schlieren, Switzerland

**Keywords:** Neuroscience, Lipids

## Abstract

The thioredoxin (TXN) system is an NADPH + H^+^/FAD redox-triggered effector that sustains homeostasis, bioenergetics, detoxifying drug networks, and cell survival in oxidative stress-related diseases. Elovanoid (ELV)-N34 is an endogenously formed lipid mediator in neural cells from omega-3 fatty acid precursors that modulate neuroinflammation and senescence gene programming when reduction-oxidation (redox) homeostasis is disrupted, enhancing cell survival. Limited proteolysis (LiP) screening of human retinal pigment epithelial (RPE) cells identified TXNRD1 isoforms 2, 3, or 5, the reductase of the TXN system, as an intracellular target of ELV-N34. TXNRD1 silencing confirmed that the ELV-N34 target was isoform 2 or 3. This lipid mediator induces TXNRD1 structure changes that modify the FAD interface domain, leading to its activity modulation. The addition of ELV-N34 decreased membrane and cytosolic TXNRD1 activity, suggesting localizations for the targeted reductase. These results show for the first time that the lipid mediator ELV-N34 directly modulates TXNRD1 activity, underling its protection in several pathologies when uncompensated oxidative stress (UOS) evolves.

## Introduction

The thioredoxin (TXN) and glutaredoxin (GRX) systems are the main glutathione effectors to modulate overall oxidative stress and sustain intracellular homeostasis, bioenergetics, and detoxifying drug networks [1]. Both oxide reductases use flavin adenine dinucleotide (FAD) as electron acceptors and nicotinamide adenine dinucleotide phosphate (NADP) H + H^+^ as proton/electron donors to reduce downstream targets that modulate protein activity and specific transcription [2]. Thioredoxin reductase 1 (TXNRD1) is required for TXN activity [3]. In addition, TXNRD1 entails a selenocysteine peptide as part of a conserved stem-loop selenocysteine insertion sequence (SECIS) of the messenger RNA (mRNA) 3’ untranslated regions (3’ UTRs). This sequence is necessary so the translation machinery inserts the modified amino acid, selenocysteine, in a specific stop codon that changes the stop signal during translation [4].

There are five different splice variants of TXNRD1 containing variable N-terminal. Two isoforms are in the nucleus and the membrane. TXNRD2 and TXNRD3—isoforms produced by gene duplication—are in the mitochondria.

Elovanoid (ELV)-N34 is an endogenously synthesized lipid mediator in neural cells from a very long fatty acid derivative (34 C) of docosahexaenoic acid (DHA, 22 C). ELV-N34 bioactivity is reflected in protection against uncompensated oxidative stress (UOS), ferroptosis, oxygen-glucose deprivation (OGD), and *N*-methyl-d-aspartate acid (NMDA) excitotoxicity in neural cells in culture [5, 6]. In addition, this bioactive lipid elicits neuroprotection after ischemic stroke in rats [5] and modulates the expression of Iduna, Sirt1, PHB, and proteins related to apoptosis, senescence gene programming [7], viral entry [8], and inflammation [9, 10].

To explore if ELV-N34 modulates TXNRD1, we used limited proteolysis (LiP) followed by mass spectrometry to determine protein interactions for ELV-N34. LiP-MS method allows us to pinpoint protein structural changes based on altered susceptibility to protease cleavages, with a resolution of 10–15 amino acids (average length of a LiP peptide). The magnitude of these changes ranges from local conformational change (e.g., through small molecule binding to an active site) to larger structural rearrangements (such as protein–protein interaction or solvent accessibility due to burial of hydrophobic areas) [11–14].

Overall, using primary human retinal pigment epithelial (RPE) cells that fully recapitulate native RPE [15] and retain features of its neuronal lineage [6, 15, 16], we demonstrate that ELV-N34 interacts with and exerts a structural configuration modification of TXNRD1. As a result, modulation of its activity takes place. By LiP analysis followed by MS, we characterize the site of lipid mediator/protein interaction. We found that ferroptosis or UOS triggers TXNRD1 structural changes that enhance its interaction with ELV-N34. Thus, our data uncover a novel regulatory mechanism of this protein other than the commonly studied transcriptional modulation and suggests its impact on protection against oxidative stress-related diseases and sustaining extended life span.

## Results

### ELV-N34 interacts with glutathione system proteins

**Fig. 1:**
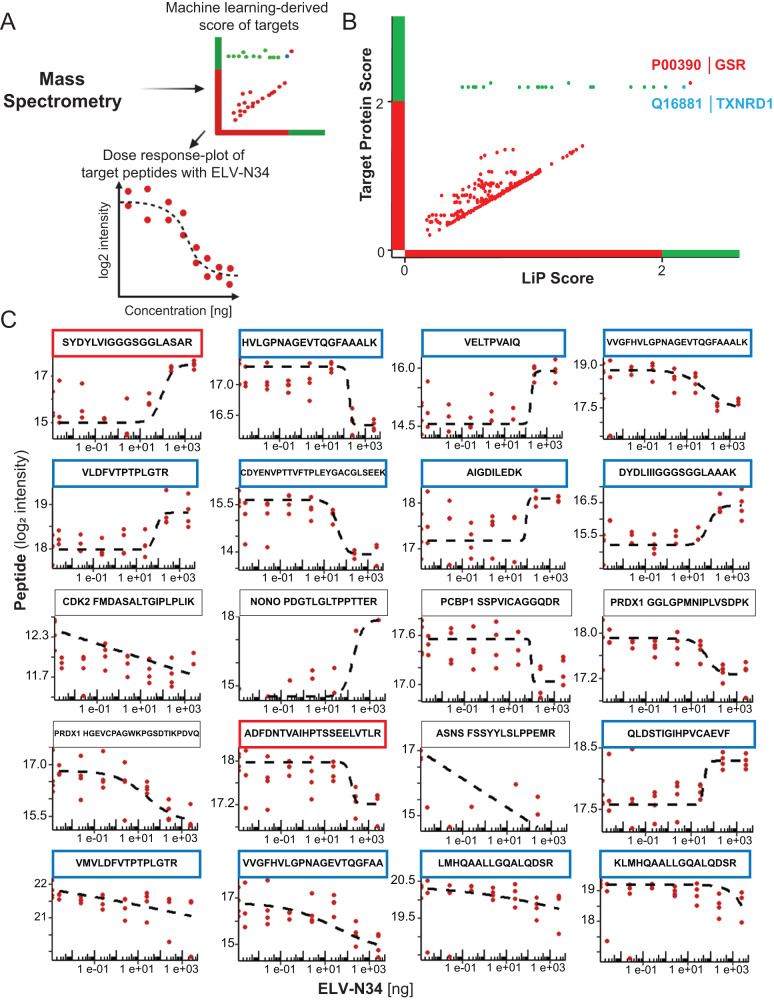
Limited proteolysis identified GSR and TXNRD1 as targets of ELV-N34. **A** Flow chart of the steps for defining targets after limited proteolysis (LiP) assay. The peptides were identified using LiP coupled to HRM-mass spectrometry for the libraries 0, and 250 ng of ELV-N34 was used to analyze via machine learning by means of Spectronaut 16 software and plotted in a graph following their target protein scores vs. LiP score. The peptides belonging to the proteins identified as targets of ELV-N34 were plotted in a 7-point dose-response curve (DMSO, 2.5 pg, 25 pg, 250 pg, 2.5 ng, 25 ng, 250 ng, and 2.5 µg). **B** Target protein Score vs. LiP score plot for the ABC cells undergoing UOS for 6 h. The intracellular content of the treated cells was exposed to vehicle or 250 ng of ELV-N34. **C** Concentration curves for each peptide. The peptide sequences are highlighted on top of their respective concentration curve. Red for GSR and Blue for TXNRD1. Other peptides corresponding to proteins that pass the Target protein but did not pass the LiP score threshold of two were plotted without highlighting (CDK2, NONO, PCBP1, PRDX1, and ASNS). Other peptides belonging to proteins identified: SERPINH1, GOT1, JPH2, PLCB3, and SPTAN1. The plots were analyzed using non-linear regression Agonist vs. Response model (black dotted line).

To assess ELV-N34 intracellular targets of human RPE cells undergoing UOS, which was induced with H_2_O_2_ plus tumor necrosis factor alpha (TNFα), the cytosol content was incubated with different concentrations of the lipid mediator (2.5, 25, 250 pg; 2.5, 25, 250 ng; and 2.5 µg) as well as with the vehicle (DMSO) and a compound that produces a known distinct effect (rapamycin) as a positive control (Fig. 1A). The assay was calibrated with rapamycin to test for specificity resulting in the predicted targets (Fig. S1). Rapamycin is a technical positive control established by Piazza et al. [12] and serves two purposes: (1) native cell lysis and conservation of cellular milieu as well as protein–protein interactions, and (2) experimental quality control for limited proteolysis. This is achieved by capturing known interactors as top-ranked candidates in the LiP-MS experiment. The peptides obtained for 250 ng of ELV-N34 were contrasted against the library that was made using DMSO (vehicle) to discard the common sequences via machine learning algorithms by Spectronaut 16 software (Fig. 1A and Tables S1–S5). The resulting differential peptides were assigned to their corresponding proteins and given a LiP score based on statistical significance and target protein score (Fig. 1B). The top-scoring proteins that were proposed to be targets of ELV-N34 were identified as Mitochondrial Glutathione reductase (GSR, UniProt ID P00390) and human cytoplasmic TXNRD1 (UniProt ID Q16881), both of them surpassing the two-score threshold for target protein and LiP (Fig. 1B). This initial identification was expanded by comparing the peptides corresponding to these two proteins for each concentration tested and plotted in a concentration curve (Fig. 1C). None of the ELV-N34 target proteins appeared in the rapamycin control (Fig. S1), suggesting that the GSR and TXNRD1 reacted specifically with the lipid mediator. Among other peptides, cyclin-dependent kinase 2 (CDK2), non-POU domain-containing octamer-binding protein (NONO), poly (rC)-binding protein 1 (PCBP1), peroxiredoxin-1 (PRDX1), asparagine synthetase (ASNS), serpin family H member 1 (SERPINH1), glutamic-oxaloacetic transaminase 1 (GOT1), phospholipase C beta 3 (PLCB3), junctophilin 2 (JPH2), and spectrin alpha non-erythrocytic 1 (SPTAN1) were represented in the library (Fig. 1C). However, their LiP score was below 2; thus, they were not considered to be main candidates (Fig. 1B, C).

Human GSR and TXNRD1 are similar proteins in structure, sequence, and function. Both utilized NADPH + H^+^ as a proton donor and FAD as an electron acceptor [1, 17], and as such, some of their sequences are highly conserved. Since only three identified peptides were linked to GSR (Figs. S2 and S3), against 15 belonging to TXNRD1 from a total of 25 top-scoring ones (Fig. S4), we asked whether these peptides were caught at low stringency conditions. The three peptides analyzed (Table S6 and Figs. S2A and S3A, B) were aligned with human cytosolic TXNRD1 using BlastP (NCBI), and two of them showed significant homology (Figs. S2C and S5). The three peptides were also highlighted in the 3D rendering obtained by X-ray crystallography available at NCBI (PDB ID#3DJJ) visualized in iCn3D viewer (Fig. S2B). The peptide of sequence SYDYLVIGGGSGGLASAR showed the most homology with TXNRD1 mapped in the 3D rendering in the FAD pocket, while the other two peptides are contiguous and mapped in the interface domain that limits the two subunits in the homodimer (Fig. S2B). The three top-scoring peptides for TXNRD1 (Table S7 and Fig. S5A) were also mapped in the interface region and FAD pocket in the 3D model (PDB ID#2ZZC, Fig. S5B). The overall LiP-MS data indicated that TXNRD1 is engaged by ELV-N34, and upon this interaction, both the NAD-binding region as well as the dimer interface are affected, suggesting that this interaction may be related to the regulation of the electron transfer to the FAD cofactor.

### Ferroptosis and UOS trigger TXNRD1 structural changes that enhance its interaction with ELV-N34

**Fig. 2:**
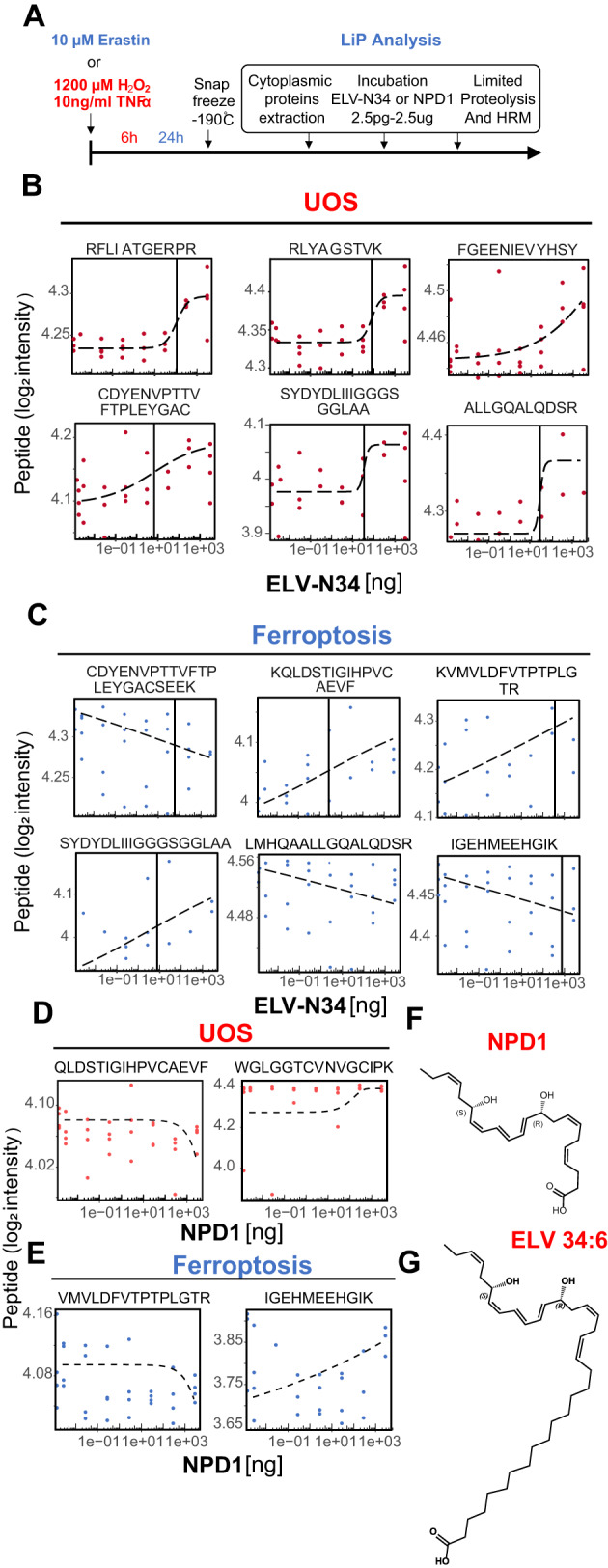
TXNRD1 is the main target of ELV-N34 under UOS or ferroptosis conditions. In a second independent experiment, only TXNRD1 peptides were top-scoring and thus selected for analysis. **A** Timeline of the treatment of the experiment. Cells were treated with 1200 µM H_2_O_2_ and 10 ng/TNFα for 6 h or 10 µM erastin for 24 h, then scraped and snap frozen. The content of the cytoplasm was incubated with 2.5 pg, 25 pg, 250 pg, 2.5 ng, 25 ng, 250 ng, and 2.5 µg ELV-N34 or DMSO (Vehicle). **B**–**E** Representative peptides corresponding to TXNRD1 resulting from cells undergoing UOS (red dots, **B**, **D**) or ferroptosis (blue dots, **C**, **E**) in the presence or absence of 7 concentrations of ELV-N34 (**B**, **C**) or NPD1 (**D**, **E**). The libraries (Figs. S8, S11, and S15–S17) were compared and plotted in a dose-dependent manner (*X*-axis is in logarithmic form). The black dotted line represents the non-linear regression model obtained using Agonist vs. Response model. The main parameters obtained by the model are displayed in Tables S6–S9. **F**, **G**, NPD1 and ELV-N34 molecule formulas.

**Fig. 3:**
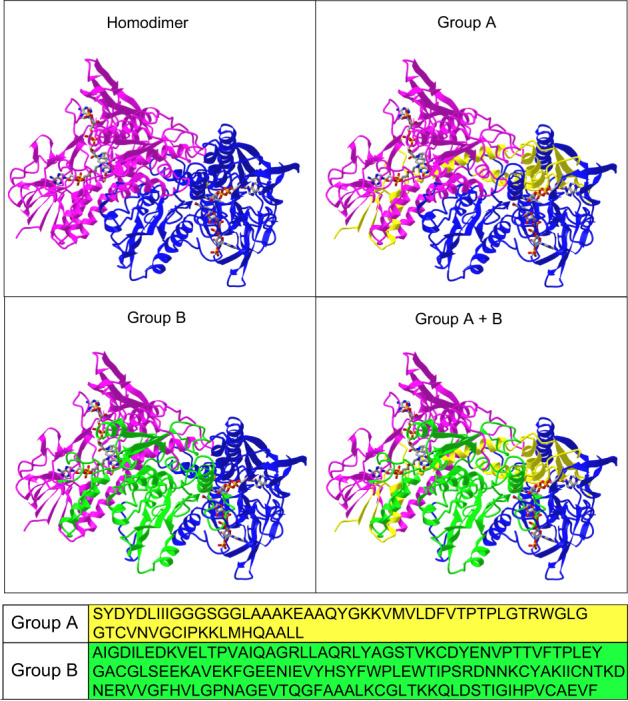
The regions affected by ELV-N34 are in close contact with the FAD pocket and the interface zone. The alignment of the peptides with the secondary structure of the TXNRD1 (Fig. S6 and Tables S6–S9) fell into two groups displayed as group A (yellow) and group B (green). The upper left quadrant shows the homodimer 3D (PDB ID: 2ZZC) rendition obtained by X-ray crystallography and available on NCBI (https://www.ncbi.nlm.nih.gov/Structure/icn3d/full.html?&mmdbid=76017&bu=1&showanno=1&source=full-feature). The right upper and the left down quadrants depict the groups A and B highlighted respectively (yellow and green) and the right down quadrant illustrates the whole zone affected by ELV-N34 that in the 3D representation appears as a continuous region.

To confirm the above findings, an additional set of independent experiments was conducted using erastin and H_2_O_2_ plus TNFα as stressors (Fig. 2A). Erastin induces the opening of the mitochondrial voltage-dependent anion channels 2 and 3 (VDAC2/3), creating an endogenous source of UOS [18], inhibits the Cysteine/Glutamate exchange via SCL7A11/SCL3A2 channel [19] to prevent the glutathione system from scavenging the mitochondrial-derived reactive oxygen species (ROS), and alters the iron-related proteins as well as induces the formation of lipid peroxides, all of which generate ferroptosis cell death [20]. The lipids used were ELV-N34 and Neuroprotectin D1 (NPD1), and the rationale behind this was to test if the two hydroxyl groups in ELV-N34, similar to those in NPD1 (Fig. 2F, G), interact with the proteins. GSR did not reach the thresholds on the second experiment, but all of the peptides from TXNRD1 were repeated for UOS or erastin-treated cell contents exposed to ELV-N34 (Fig. 2B–D; Tables S8–S12), confirming the specificity of the interaction (Fig. 2B, C, Fig. S1, and Tables S8 and S9). Moreover, two of those peptides were displayed in the library obtained by comparing DMSO and NPD1 (Fig. 2D, E and Tables S10 and S11), suggesting that the portion that carries the hydroxyl groups interacts with the TXNRD1. The NPD1 concentration curves showed negative or large ED50s when their non-linear fit models were run (Peptides 1 to 4; Tables S10 and S11) and different from the same peptide when ELV-N34 was applied. When all the peptides were aligned into the cytoplasmic TXNRD1 sequence, the peptides 1 to 4 from NPD1 concentration curves (Fig. 2D, E and Tables S10 and S11) matched with corresponding sequences 2, 3, and 6 (Table S9) and the fourth plot in Fig. 1C (Fig. S6). The sequences from all the peptides identified robustly fell into two regions of the protein named Group A and B (Fig. 3 and Fig. S6) of the known isoforms of human cytoplasmic TXNRD1 (Table S13), except for the peptide AAETDLPVVFVK. Notably, this sequence only matched with isoforms 2, 3, and 5 (NP_003321.3, NP_001248374.1, NP_001087240.1), ruling out v1 and v4 (NP_877393.1, NP_001283751; Tables S8 and S9 and Fig. S6). The localization of Groups A and B in the 3D rendering of the human cytoplasmic TXNRD1 showed that the interaction with ELV-N34 was localized in the FAD and interface domains of the homodimer (Fig. 3). Based on this model, the structural proximity of the sequences obtained with the LiP technique points to a probable allosteric action of the lipid mediator that could be involved in the modulation of the electron transfer and/or exit from the oxidized FAD, and thus altering the activity of the cytoplasmic TXNRD1.

TXNRD proteins are reductases that reduce the effector protein coupled to them, TXN, which instead reduces disulfide bonds of their targets. For the TXNRD1 to reduce TXN, electrons should be transferred to their FAD cofactor [21]. The alignment of all peptides showed a consistent occurrence when ELV-N34 was added and conveyed in two main regions that seemed structurally continuous in the 3D model (Fig. 3, Fig. S7, and Tables S8 and S9). The regions interlocked enclosed the FAD pocket (FAD domain) and the interface between the two monomers in the homodimer, suggesting that the lipid may favor or limit the exit of the reduced FAD and thus putatively alter the transfer of electrons. In any case, the NADP domain was unaltered in the interplay.

The response elicited by NPD1 was partial; only some of the peptides appeared in the library, leading to the idea that the saturated part of the lipid is necessary for the full activity of ELV-N34.

### ELV-N34 modulates TXNRD1 activity

**Fig. 4:**
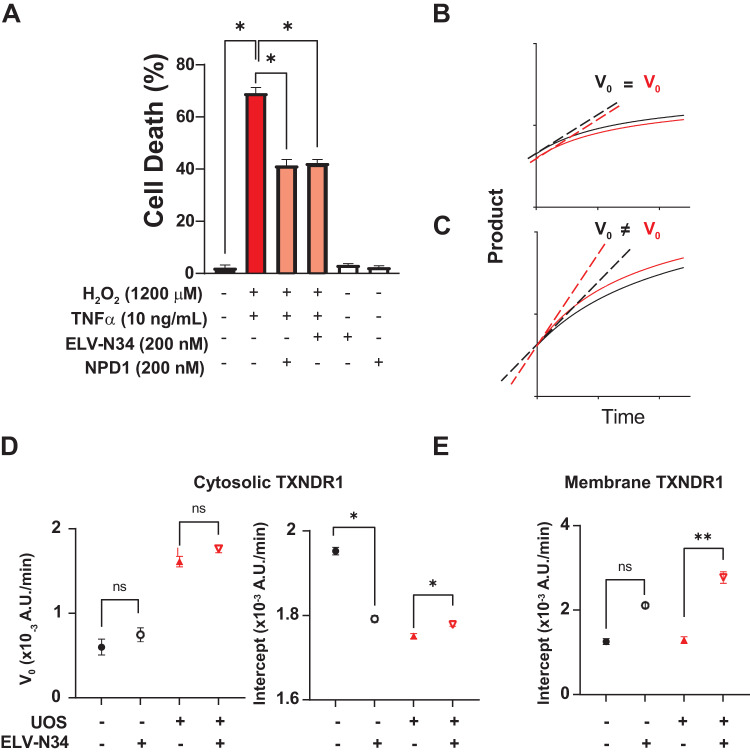
ELV-N34 alters the activity of membrane and cytosolic TXNRD1 under UOS conditions. **A** ABC cells exposed to 1200 µM H_2_O_2_ and 10 ng/ml TNFα are protected by 200 nM of NPD1 or ELV-N34. Bars represent the mean +/− SEM. *p < 0.05. **B**, **C** Cartoon to explain how the velocity Vo and the intersections with the Y axis (intercepts) differences were assessed to determine the data points in D and E and Fig. 7A, B from original data in Fig. S11 (**D**), Fig. S10 (**E**), to show differences in modulation. **D**, **E** The TXNRD1 obtained from the cytosolic fraction of ABC cells undergoing UOS showed same Vo (**D**) and different intercept (**E**). **E** The membrane TXNRD1 shows differences in Vo in ABC cells undergoing UOS in the presence of ELV-N34. Circles represent naïve cells in the presence (open) or absence (full) of ELV-N34. Triangles depict cells undergoing UOS in the presence (open, pointing down) or absence (full, pointing up) of ELV-N34. The bars show the SEM and the mean of triplicates. *p < 0.05; **p < 0.001. Vo was obtained using linear regression modeling of the first data points of the curves and ANCOVA to compare the slopes.

ELV-N34 interaction with TXNRD1 induced structural changes in the protein compatible with allosteric modulation of the activity. The conformational alterations suffered by the homodimer may modify the rate at which the transfer of electrons to FAD occurs or even the exit of the oxidized acceptor from the pocket, modulating its enzymatic reduction rate. The structural changes were observed in cells that were not UOS or ferroptosis, and the peptides that emerged from the comparison between DMSO and ELV-N34 showed common peptides with stressed cells but also unique ones (Table S12). To assess the changes in the TXNRD1 enzymatic activity induced by ELV-N34, ABC cells were treated with 2000 µM H_2_O_2_ and 10 ng/ml TNFα for 2 h or alternatively with 100 µM of erastin for 24 h in the presence or absence of ELV-N34. The cells were lysated and tested for total TXNRD1 activity (Fig. S8). Fractions obtained by ultracentrifugation that were enriched in nuclei (Fig. S9), membrane (Fig. S10) or cytosolic (Fig. S11) were also assessed for TXNRD1 activity (Fig. 4) was performed. UOS induced a steep percentage of cell death that was protected by ELV-N34 and NPD1 (Fig. 4A). The activity of TXNRD1 was measured in the total cell lysate (crude extract), cytosolic, membrane, and nuclear fractions of cells undergoing UOS or control in the presence or absence of 200 nM ELV-N34. The reduction activity of TXNRD1 was determined using the artificial substrate DTNB through the oxidation of the NADPH + H^+^ by the TXNRD1. Two moles of 5-thio-2-nitrobenzoic acid (TNB) are formed for every 1 mole of NADPH + H^+^ oxidized, releasing color to the medium that absorbs the light at 412 nm wavelength. The colorimetric reaction was measured every 5 min for 2 h. The plots of product vs. time were subjected to non-linear regression to obtain the curve (Figs. S10 and S11). The initial 15 min of the curve were then analyzed by linear regression, and the slope or initial velocity (Vo) and the intersect (b) were compared between the curves (Fig. 4B, C). Either a difference in Vo or in the intersection between curves was noted as differential activity of the enzyme. Cytosolic TXNRD1 activity differed in both naïve and stressed cells when they were in the presence of ELV-N34 vs. the control (Fig. 4D and Fig. S11). In addition, the enzyme in the membrane fraction showed differences in the reduction of DNTB into TNB only in cells undergoing UOS when compared ELV-N34 vs. vehicle (Fig. 4E and Fig. S10).

**Fig. 5:**
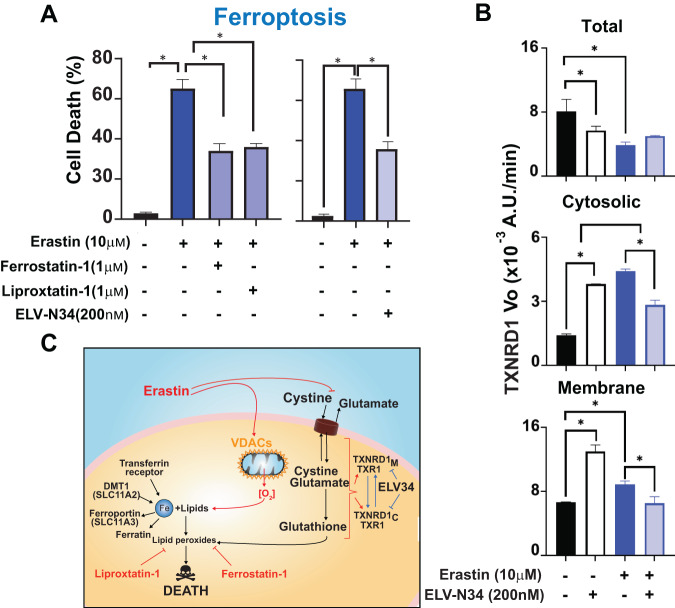
TXNRD1 Vo is modulated by ELV-N34 during ferroptosis. **A** Ferroptosis by erastin in ABC cells is confirmed by the protection of 1 μM ferrostatin-1 and 1 μM liproxstatin-1 inhibitors (left panel). The addition of 200 nM ELV-N34 in the same conditions prevents ferroptosis (right panel). **B** ABC cells incubated with erastin for 24 h depict a modulation of the cytosolic (Fig. S13) membrane (Fig. S14) and total (Fig. S12) TXNRD1 Vo. The bars show the SEM and the mean of triplicates. *p < 0.05. **C** Site of action of erastin, ferrostatin-1, liproxstatin-1, and TXNRD1 plus ELV-N34. Vo was obtained using linear regression modeling of the first data points of the curves and ANCOVA to compare the slopes.

Erastin, an inducer of ferroptosis, blocks the interchange of cystine/glutamate by SLC7A11 [19] and induces a mitochondrial-derived intracellular increase in ROS [18] (Fig. 5C). Ferrostatin-1 and liproxstatin-1 protect the cells from ferroptosis by preventing the formation of lipid peroxides (Fig. 5A, C). ELV-N34 prevents cell death by ferroptosis to the same extent as ferrostatin-1 and liproxstatin-1 (Fig. 5A). ABC cells treated with erastin showed a different TXNRD1 profile of activity than those cells treated with H_2_O_2_ even though they shared a great part of the spatially altered peptides (Figs. 2–4 and Tables S7–S9 and S12). Because the stress induced by erastin and H_2_O_2_ is different, the profiles obtained in both treatments differed. One possibility is that the activity in each case is produced by stress-specific movement of the peptides within the protein that enhance or preclude the electron transfer. In the crude extract, erastin induced a decrease in the overall initial enzymatic velocities (Fig. 5B, top panel, and Fig. S12). The initial velocity of the cytosolic enzyme was lower than in the membrane. ELV-N34 increased the Vo of TXNRD1 in the cytosolic and membrane-enriched fractions in control but reduced its velocity in cells undergoing ferroptosis, suggesting that both enzymes could be coming from the same source (Fig. 5B and Figs. S13 and S14).

Ferroptosis and UOS condition peptides were slightly different [18]. The activity confirmed these observations. The subfractions showed a correspondence between cytosolic TXNRD1 and membrane-enriched fraction enzyme. The membrane-enriched fraction may contain not only plasma but also other small or disaggregated membranous organelles like vesicles from Golgi and endoplasmic reticulum (ER), among others. The activity was steady in the membrane-enriched pellet, suggesting a pool of the enzyme we call TXNRD1_M_, which may be located in one or more of these subcellular organelles, interchange enzyme with the cytosolic pool TXNRD1_C_ (Fig. 5C).

**Fig. 6:**
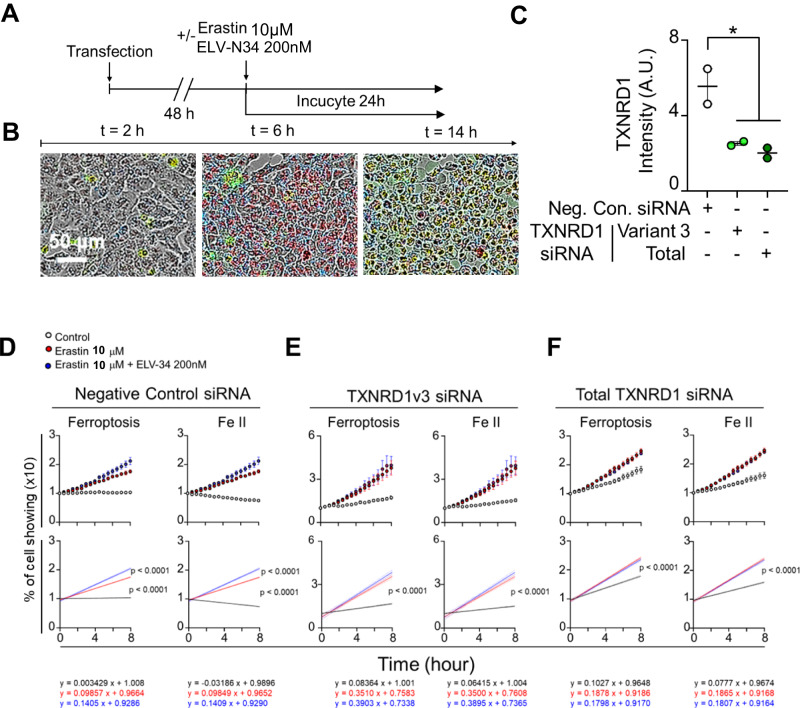
ELV-N34 lost activity when TXNRD1 isoforms 2/3 were silenced. **A** Timeline of ferroptosis real-time recording of TXNRD1 isoforms 2/3 knocked-down cells**. B** Representative images of cells at 2, 6, and 14 h after treatment (linear period). Transfected cells showed tracer (blue), dead cells were permeable to Sytox green (green) and ferrous ion accumulation is shown in red (FeII). **C** Quantification of capillary western blot when ABC cells were transfected with siRNAs targeting total and isoforms 2 and 3 TXNRD1 along with a universal negative control. Pictures of the whole capillary western blot run and the total protein stain are depicted in Fig. S21. **D**–**F** Negative control (**D**), isoforms 2/3 (**E**), and total (**F**) knocked-down human RPE cells that showed Sytox green and accumulation of FeII (ferroptosis, left top panels), and accumulation of FeII (right top panels). The alignment of transcripts and siRNA and their correspondence with the protein Isoforms is depicted in Fig. S20 and Table S13. The numbers plotted are the mean of 8 images 30 min, one image per well with the standard error of the mean. To calculate the differences between treatments, a linear regression model was applied. The curves were compared using ANCOVA.

To confirm the interaction between ELV-N34 and TXNRD1, we selectively knocked-down isoforms 2 and 3 and tested the formation of ferrous cation (FeII), indicative of ferroptosis and the simultaneous incorporation of Sytox green, a dye that only enters the cell upon death-induced permeability via real-time imaging (Fig. 6A, B). A blast-p search (NCBI) showed that 100% of the nucleotides in transcript variants 4 and 5 (Tables S1 and S13) correspond to TXNRD1 cytoplasmic isoforms 2 and 3 protein (Tables S2 and S13), and only 99% of this transcript was covered in isoforms 5 and 2 (Table S7). Silencing of TXNRD1 protein isoforms 2 and 3 (Fig. 6C), corresponding to transcript variants 4 and 5, showed an abrogation of ELV-N34 protection when cells were treated with erastin 100 μM (Fig. 6D, E). Similarly, the total TXNR1 silencing tagging exons common to all the variants showed similar results as the protein isoforms 2 and 3 knock-down in comparison with the negative control (Fig. 6D, F).

**Fig. 7:**
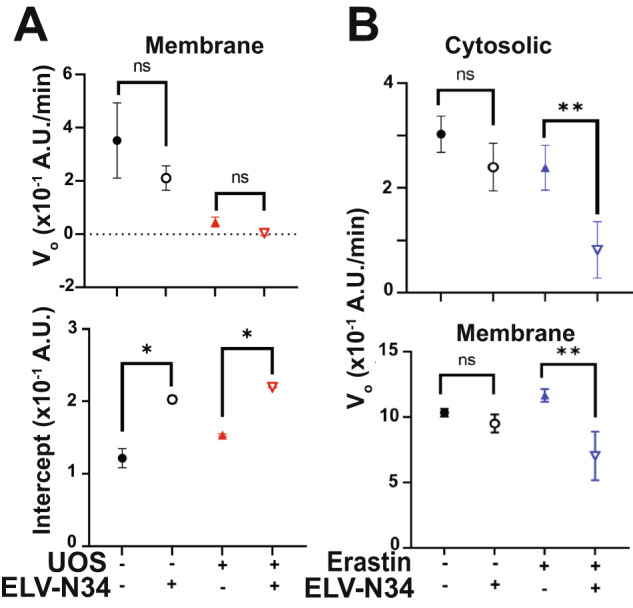
ELV-N34 modulates membrane and cytosolic TXNRD1 activity. **A**, **B** 250 ng of ELV-N34 was added to membrane (**A**, **B**) or cytosolic (**B**) fractions of ABC cells exposed to UOS (**A**) or erastin (**B**) vs. naive (computed from Figs. S15 to S17). Based on the activity curves of TXNRD1 Vo, intercept was obtained for each triplicate using linear regression of the first 15 min of recording and plotted. Circles represent naïve cells in the presence (open) or absence (full) of ELV-N34. Triangles depict cells undergoing UOS in the presence (open, pointing down) or absence (full, pointing up) of ELV-N34. Red symbols for UOS, blue symbols for ferroptosis. The bars show the SEM and the mean of triplicates. *p < 0.05; **p < 0.001. Vo was obtained using linear regression modeling of the first data points of the curves and ANCOVA to compare the slopes.

The addition of ELV-N34 to the membrane and cytosolic enriched fractions of cells undergoing UOS induced an increase in initial intersect only in the membrane fraction, showing an activation or increment in the rate of conversion of DNTB coincident with the effect of ELV-N34 in the intact cell (Fig. 7A and Fig. S15). Similarly, ELV-N34 slows down the activity of the enzyme in both fractions obtained from cells treated with erastin (Fig. 7B and Figs. S16 and S17). Overall, these results confirm the observations made previously by LiP analysis and provide evidence for the first time that a lipid mediator is involved in the allosteric modulation of the activity of TXNRD1.

ELV-N34, a derivative of omega-3 FA DHA, elicits pro-homeostatic activity via activation of pathways that enhance survival and prevent inflammation, senescence, and cell death under damaging conditions. As elovanoids are non-conventional ligands that impinge on cell fate decision pathways, we adopted a non-conventional approach that uncovered the primary targets of these bioactive lipids. Limited proteolysis followed by high-resolution mass spectrometry (LiP-HRM), taking advantage of the structural changes that proteins undergo in the presence of interacting molecules, reveals ELV-N34 as a ligand and modulator of TXNRD1. Therefore, the pleiotropic responses exerted by elovanoids use a few sites of action for signal tuning and amplification, leading to crosstalk between pathways.

## Discussion

ELV-N34, derived from omega-3 fatty acid precursors, elicits pro-homeostatic activity, modulates neuroinflammation and senescence gene programming, and sustains cell survival under pathological conditions [5–7]. Since ELVs are non-conventional ligands that target cell fate decision pathways [9], we adopted a non-conventional approach that uncovered the primary targets of ELV-N34. LiP-HRM reveals ELV-N34 as a ligand and modulator of TXNRD1. Therefore, the pleiotropic responses exerted by ELVs likely engage this effect for signal tuning and amplification, leading to crosstalk between pathways.

Silencing of protein isoforms 2 and 3 yielded a small decrease (16%) of the total protein targeted, as shown by capillary western blot (Fig. 6C). The siRNA tagging Exon 10, which is common to all variants, showed a 46% difference (Fig. 6C). The small decrease observed in the isoforms 2 and 3 knock-down suggests a modest representation among the total variants. However, the fact that the total TXNRD1 silencing led to similar results advocates in favor of the selectivity of the interaction between ELV-N34 and this variant.

The relevance of TXNRD1 has been increasingly acknowledged in recent years and has become the focus of understanding fundamental homeostatic cell functions as well as therapeutic approaches for cancer and other pathologies. However, the understanding of the regulation of its activity has remained stagnant. Here, for the first time, we uncover that a lipid mediator interacts and exerts a structural configuration modification on TXNRD1 that leads to the modulation of its activity. TXNRD1 control pathways of cell viability, ferroptosis, ER stress response, and mitochondria renewal, among other changes, are triggered by redox states [22, 23]. TXNRD1 is coupled to TXN in the TXR complex. TXR targets several important metabolic and cell survival-related pathways, including the Thioredoxin interacting protein (Txnip), an alpha-arrestin type protein involved in cancer suppression and the regulation of glucose uptake [22]. However, in this case, the activity is located mainly in membrane fractions that contain parts of the ER as well as the plasma membrane. The ER is the site of synthesis and maturation of membrane and secretory proteins in eukaryotic cells. The ER is the home of the protein disulfide isomerase (PDI) family, which regulates the formation, isomerization, and disassembly of covalent bonds between cysteine residues to ensure correct protein folding is competently transported and protein unfolding of defective gene products for ER-associated degradation (ERAD). Among these PDIs, there are five groups in the thioredoxin-related transmembrane (TMX) protein family: TMX1-5. The TMX basal state is reduced, and as ER stress overcomes, the pool becomes oxidized. Reductases may get into play to restore the pool of active TMX so the ERAD can proceed [23]. The results indicate that TXNRD1 from the membrane and cytosol could be the reductase from the glutathione system [24] that switches the TMX on-off at the different locations, including the ER, connecting its function with protein unfolding with many pathologies, including neurodegenerative diseases.

ELV-N34 is synthesized from omega-3 fatty acid precursors upon demand and modulates TXNRD1 activity. Together, our data point to a novel endogenous mechanism other than transcriptional modulation that may divert the activity of the TXN complex towards homeostasis when needed, responding to redox changes, protecting against oxidative stress-related diseases, and sustaining an extended life span.

### Limitations of the study

LiP-HRM allowed the identification of TXNRD1 as a target of ELV-N34. However, the technique possesses limitations, some of which are mentioned above. In addition, the available 3D depiction was produced on the available crystallographic rendering of TXNRD1, which was synthesized for a study on *E. coli* [25]. TXNRD1, as a selenoprotein, depends on complex machinery to ensure that the seleno-modified amino acid in the N-terminal is correct [26]; this mechanism is not present in the protein machinery of *E. coli*. Thus, the 3D rendering is approximate yet not completely accurate. The gold standard techniques to determine structural changes are X-ray crystallography and MRI, both of which are ongoing in our lab. Because of the peptides defined by LiP-HRM, the possible isoforms were narrowed down to 2 or 3 (2, 3, or 5, or a mix of the three). Isoforms 2 and 3 only differ on two amino acids, and the distinction via specific siRNAs or primers via RT-PCR is very difficult. In future directions, we will target these two isoforms with direct mutagenesis to determine not only which one/s are regulated by ELV-N34 with greater precision but also their subcellular localization.

## Methods

### Cell preparation and treatments

Human RPE cell pellet containing one hundred million cells plus ELV-N34 Na or Neuroprotectin D1 (NPD1-Na) in DMSO were used for LiP-compound dose response combined with HRM mass spectrometry assay. ELV-N34 is 16Z,19Z,22 R,23E,25E,27Z,29S,31Z)-22,29-dihydrox ytetra-triaconta-16,19,23,25,27,31-hexaenoic acid [5, 6, 10]. For apoptosis measurement, cells were plated in a 500,000 cell per well density in six-well plates. The next day, the medium was changed to low serum for 8 h and then treated with 1200 μM H_2_O_2_ and 10 ng/ml TNFα in the presence or absence of 200 nM ELV-N34. The treatment was left for 24 h, the time required for the shrinking of the nuclei to develop. After this period, the cells were fixed, and the apoptotic cells were counted [15]. Erastin was used at 10 μM to induce ferroptosis when the conditions allowed for a long incubation (24 h); otherwise, the concentration used for short-term experiments was 100 μM. Liproxstatin-1 and ferrostatin-1, two ferroptosis inhibitors, were used to confirm the cause of cell death. The cells undergoing apoptosis or related cell death and ferroptosis were differentiated by adjusting the size and brightness of the nuclei.

The experiment was repeated twice to obtain the results described in this manuscript. LiP Lysis Buffer (native conditions, HEPES pH = 7.5) was added to the cell pellet. Pellet suspension was homogenized by aspiration/ejection through a 27 G BD PrecisionGlide™ syringe needle (L 1 1/4 in) 10 times. After pelleting cell debris, lysate was aliquoted into 100 µg per tube to perform the 7-step concentration curve (2.5, 25, 250 pg; 2.5, 25, 250 ng; and 2.5 μg) of ELV-N34, NPD1, and the negative and positive controls (DMSO and rapamycin, correspondingly). Digestion with proteinase K (protein: protease ratio of 100:1) was performed and stopped by rapidly heating to 98 °C coupled with the subsequent addition of sodium deoxycholate to a final concentration of 5%. Samples were reduced with 5 mM tris-(2-carboxyethyl)-phosphine for 1 h at 37 °C and alkylated with 20 mM iodoacetamide for 30 min at room temperature in the dark. Subsequently, samples were digested for 2 h with Lysyl (WAKO) and overnight with trypsin (Promega), both at 37 °C and at a protein: protease ratio of 100:1.

### Clean-up for mass spectrometry

Peptides were desalted using a C18 MicroSpin plate (The Nest Group) according to the manufacturer’s instructions and dried down using a SpeedVac system. Peptides were resuspended in 20 µl LC solvent A (1% acetonitrile, 0.1% formic acid (FA)) and spiked with Biognosys’ iRT kit calibration peptides. Peptide concentrations were determined using a UV/VIS Spectrometer (SPECTROstar Nano, BMG Labtech).

### HPRP fractionation

For HPRP fractionation, a combined peptide pool was generated containing equal volumes of all samples. Ammonium hydroxide was added to a pH value > 10. The fractionation was performed using a Dionex UltiMate 3000 RS pump (Thermo Scientific) on an Acquity UPLC CSH C18 1.7 µm, 2.1 × 150 mm column (Waters). A non-linear (1–40% solvent B) 30 min gradient was run with 30-sec micro fractions sequentially pooled into a total of 15 fractions. Solvents used were A: 20 mM ammonium hydroxide in H_2_O, B: 100% acetonitrile. Fractions were dried down and resolved in 20 µl solvent A spiked with Biognosys’ iRT kit calibration peptides. Peptide concentrations were determined using a UV/VIS Spectrometer (SPECTROstar Nano, BMG Labtech).

### Shotgun LC-MS/MS for spectral library generation

For shotgun LC-MS/MS measurements, 2 µg of peptides were injected into an in-house packed C18 column (Dr. Maisch ReproSil Pur, 1.9 µm particle size, 120 Å pore size; 75 µm inner diameter, 50 cm length, New Objective) on a Thermo Scientific Easy nLC 1200 nano-liquid chromatography system connected to a Thermo Scientific^TM^ Q Exactive^TM^ HF mass spectrometer equipped with a standard nano-electrospray source. LC solvents were A: 1% acetonitrile in water with 0.1% FA; B: 15% water in acetonitrile with 0.1% FA. The non-linear LC gradient was 1–55% solvent B in 120 min, followed by 55–90% B in 10 sec, 90% B for 10 min, 90% - 1% B in 10 s, and 1% B for 5 min. A modified TOP12 method from Kelstrup et al. was used [27]. Quality controls were produced using rapamycin as a calibration standard (Fig. S18) and specificity using the ABC cells, and the sample libraries were obtained from different concentrations of ELV-N34, NPD1, and DMSO under oxidative stress conditions and ferroptosis (Tables S1–S5) were normalized accordingly (Fig. S19).

### Database search of shotgun LC-MS/MS data and spectral library generation

Shotgun LC-MS/MS runs of pool fractions were analyzed using search engine Spectronaut 16 (Biognosys, Zurich, Switzerland); the false discovery rate on peptide and protein levels was set to 1%. Data were searched against a human UniProt/fasta database (*Homo sapiens*, 2019-01-07), allowing for two missed cleavages and variable modifications (N-term acetylation, methionine oxidation). The results of this search were combined with previously generated LiP shotgun LC-MS/MS search data and used to create a hybrid spectral library using Spectronaut 16 software (Biognosys).

### HRM mass spectrometry acquisition

For DIA LC-MS/MS measurements, 2 µg of peptides per sample were injected into an in-house packed C18 column (Dr. Maisch ReproSil Pur, 1.9 µm particle size, 120 Å pore size; 75 µm inner diameter, 50 cm length, New Objective) on a Thermo Scientific Easy nLC 1200 nano-liquid chromatography system connected to a Thermo Scientific^TM^ Q Exactive^TM^ HF mass spectrometer equipped with a standard nano-electrospray source. LC solvents were A: 1% acetonitrile in water with 0.1% FA; B: 15% water in acetonitrile with 0.1% FA. The non-linear LC gradient was 1–55% solvent B in 120 min, followed by 55–90% B in 10 sec, 90% B for 10 min, 90% - 1% B in 10 s, and 1% B for 5 min. A DIA method with one full-range survey scan and 22 DIA windows was used.

### HRM data analysis

HRM mass spectrometric data were analyzed using Spectronaut™ Pulsar X software (Biognosys). The false discovery rate on protein and peptide levels was set to 1%, and data were filtered using row-based extraction. The assay library (protein inventory) used was the experiment-specific library. The HRM measurements analyzed with Spectronaut were normalized using local regression normalization [28]. The *q*-value filtered data set was used for analysis.

### LiP and HRM LC-MS/MS protein profiling

ABC cells [15] were treated with 1600 µM H_2_O_2_ and 10 ng/ml TNFα for 6 h before harvesting and snap freezing in liquid nitrogen. Cells were disrupted using Biognosys proprietary optimized native lysis protocol. ELV-N34 (2.5 pg–2.5 μg per experiment, 7 steps), positive control Rapamycin or DMSO were added in 4 replicates each to lysate aliquots containing equal total protein amounts. LiP was performed using proteinase K, quenching of the reaction, and then applying trypsin digestion followed by C18 column purification of peptides. Then, Hyper Reaction Monitoring (HRM™) mass spectrometry was performed, allowing the acquisition of all detectable peptide signals in a comprehensive HRM map. The generation of a comprehensive and sample-specific spectral library was carried out using High pH-reversed phase (HPRP) fractionation of a sample pool generated from all samples (15 fractions). Shotgun LC-MS/MS was done on all fractions, and spectral library generation and quality control were performed. HRM LC-MS/MS protein profiling consisted of recording HRM data, data extraction and analysis, QC metrics, construction of unsupervised hierarchical clustering, and statistical testing (*t*-tests) for the identification of differentially expressed peptides between the conditions. The stratification of candidate peptides was conducted through dose-response curve fitting and ranking of protein candidate targets using LiP score. Pairwise statistical analysis on peptide level between lysates treated with various concentrations of ELV-N34, 2 μM Rapamycin or DMSO using 4 technical replicates.

### TXNRD1 activity assay

ABC cells were cultured following standard procedures [15] and treated with 2000 µM H_2_O_2_ and TNFα 10 ng/ml in the presence or absence of 200 nM ELV-N34 for 2 h in the T75 flask after reaching total confluency and polygonal shape. ABC cells are highly resistant to oxidative stress and ferroptosis, and curves of sensitivity have been done elsewhere [15]. Cells were scraped and incubated into fractionation buffer to proceed to subcellular fraction separation by ultracentrifugation using the protocol described elsewhere [29]. The pellets corresponding to nuclear, mitochondrial, and membrane enriched fractions were resuspended in working buffer Assay Buffer (100 mM Potassium phosphate pH 7.0 containing 10 mM EDTA) to assay TXNRD1 activity using Thioredoxin Reductase Assay Kit (Sigma, Ronkonkoma, NY cat# CS0170) that utilizes the artificial substrate 5,5’-Dithiobis(2-nitrobenzoic) acid (DTNB), which is reduced directly into 5-thio-2-nitrobenzoic acid (TNB) by TXNRD1 without Thioredoxin component of the TXN complex. The production of TNB was measured by absorbance at 412 nm. The cytosolic fraction (supernatant at 100,000 g) was concentrated 5 times their volume with Amicon Ultra 10 KDa centrifugal filters to be used as TXNRD1 source in the activity assay. To obtain the total activity, crude extract was used. Crude extract was obtained by homogenizing the cells in assay buffer using a syringe with a 25-gauge needle. TXNRD2 and TXNRD3 contribution was taken off by assessing the activity using a specific inhibitor of TXNRD1 and subtracting it from the main measurement.

### Sequences and data analysis

Distance in heat maps was calculated using the “Manhattan” method, with the clustering employing “ward. D” for both axes. Dose-response curves were calculated using *drc* package in R using a Four-Parameter Log-Logistic Function. General plotting was done in R using *ggplot2* and *shiny* packages.

PDB structure 2ZZC was employed as a structural model to visualize these three LiP peptides.

Protein 3D structures were obtained from the protein data bank (https://www.rcsb.org/) and visualized using Pymol software or iCn3D (2ZZC(MMDB) in iCn3D (nih.gov)) [30]. Candidate peptides were ranked according to Biognosys (Zürich, Switzerland) optimized LiP score. Target proteins were ranked based on the maximal peptide LiP score. Peptides were aligned using BlastP [31]. LiP-MS data analysis was carried out as described in Piazza et al. [12]. Differentially regulated peptides were identified based on comparison between the highest dose and vehicle control conditions using one sample two-sided *t*-test followed by multiple testing corrections to obtain FDR-adjusted *q*-value for each peptide [32].

For TXNRD1 activity curves analysis, non-linear regression was used to fit product vs. time curves and linear regression to obtain initial velocity (Vo) for the first 5 timepoints of the curve. The fitting curves were compared by ANCOVA, ANOVA, and Tukey’s HSD test; multiple comparisons were performed using GraphPad 9.0 (Prism, Boston, MA).

### Silencing of TXNRD1 and real-time imaging of ferroptosis

Human RPE cells were seeded in a density of 25 K cells per well in 96-well Sartorius Imagelock plates (Sartorius, Bohemia, NY, #BA-04855) with the transfection complexes. The transfection complexes containing 100 pmol/ml siRNA (Table S1), 10 pmol/ml siRNA conjugated with AlexaFluor 647 (Qiagen, Germantown, MD, #1027295) and Lipofectamine 2000 (ThermoFisher, Waltham, MA #11668-027) were prepared following the manufacturer directions. Custom siRNA for TXNRD1 isoforms 2/3 was designed using GeneAssist™ Custom siRNA Builder from Thermo Fisher (Tables S2 and S13 and Fig. S20. The siRNA was confirmed to sit on transcripts variants 4 and 5 corresponding to Isoforms 2 and 3 as per NCBI HUGO gene ID HGNC12437 (Table S13 and Fig. S20). The cells were incubated for 48 h and treated with erastin 100 μM, ELV-N34 200 nM, and FeII staining (BioTracker Ferro Orange Live Cell Dye EMD Millipore, Burlington, MA, #SCT 210-175) for the start of the 24 h recording. Experiments that allowed longer incubation times were performed using erastin (10 μM), while the short-term incubations were scaled up in erastin to 100 μM observe rapid effects. The cells were recorded every 30 min, one picture per well, 8 wells per each of the two experiments. The analysis was done using only the transfected cells (recorded in blue, Fig. 6B). Linear regression and ANCOVA were applied to determine the slope and significant differences between curves. Silencing was confirmed using capillary western blot by means of Jess (ProteinSimple/Bio-Techne, San Jose, CA) following the manufacturer’s directions and total protein to standardize. The antibody used was Proteintech, Rosemont, IL, cat #11117-1AP. Unmodified capillary western blots are depicted in Fig. S12, along with the total protein stain.

### Supplementary information

Reproducibility Checklist

Table S1

Table S2

Table S3

Table S4

Table S5

Table S6

Table S7

Table S8

Table S9

Table S10

Table S11

Table S12

Table S13

Figures S1-S21

Original Data File

## Data Availability

All the data supporting the findings of this study are available from the corresponding author on reasonable request.
