# Supplementary material for: Elovanoid-N34 modulates TXNRD1 key in protection against oxidative stress-related diseases
Source: Cell Death Dis. 2023 Dec 13;14(12):819. doi: 10.1038/s41419-023-06334-6 (PMC10716158; doi:10.1038/s41419-023-06334-6)
Supplement: Supplementary file 15 — Figures S1-S21 [file 41419_2023_6334_MOESM15_ESM.pdf]

Supplementary Material

Elovanoid-N34 modulates TXNRD1 key in protection against oxidative stress-related diseases

Jorgelina M. Calandria, Surjyadipta Bhattacharjee, Sayantani Kala-Bhattacharjee, Pranab K. Mukherjee, Yuehan Feng, Jakob Vowinckel, Tobias Treiber and Nicolas G. Bazan*

*Correspondence: nbazan@lsuhsc.edu.

Running title: Elovanoid-N34 homeostatic switch modulates TXNRD1

Contents:

Figs. S1-S21

SUPPLEMENTARY FIGURES

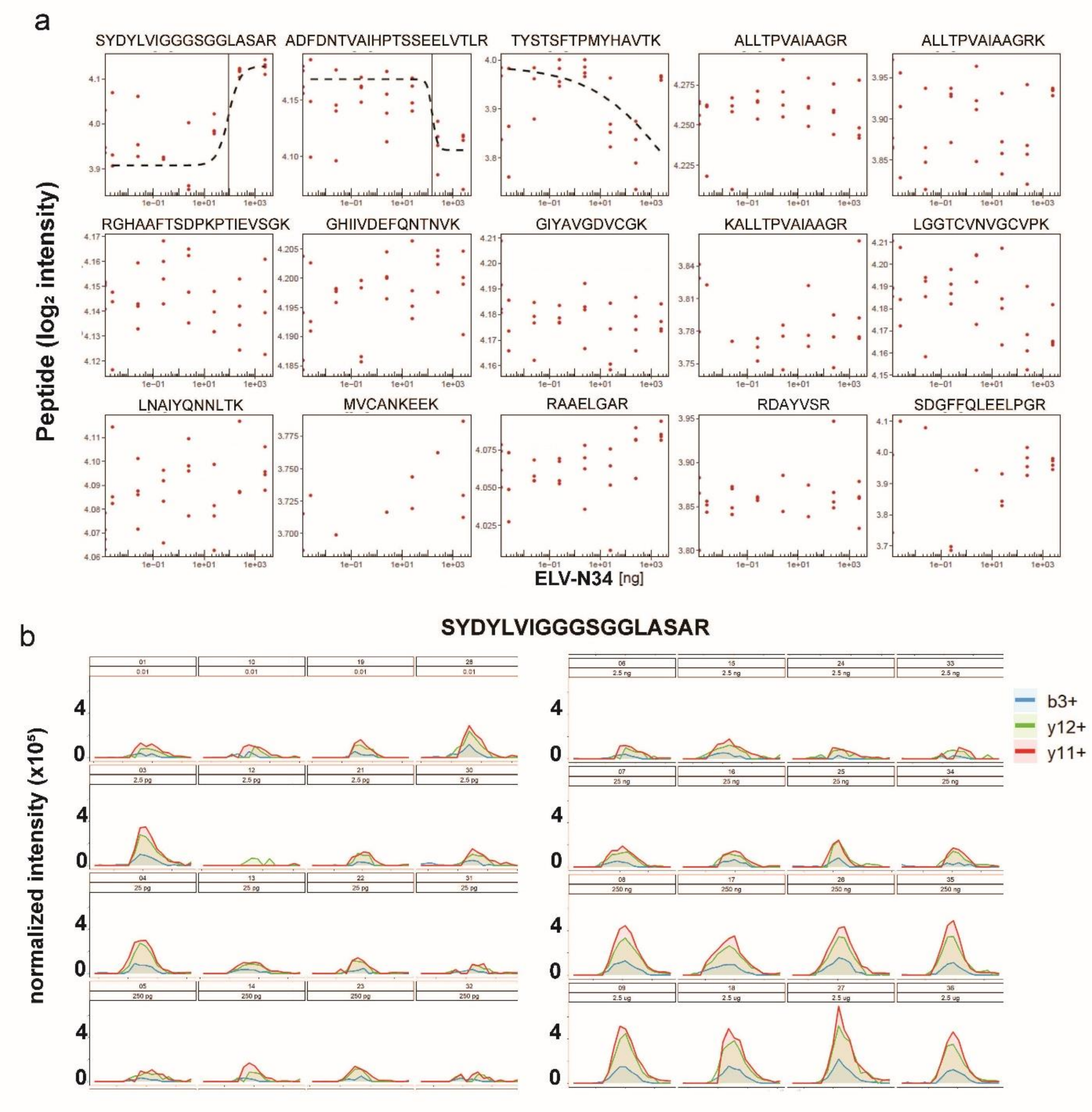

Fig. S1. Principal peptide from Glutathione reductase. a, Glutathione reductase, mitochondrial (GSR; P00390) peptides that appeared in the UOS + ELV-N34 LiP comparison. From the top 15 quantified GSR peptides in the comparison DMSO and ELV-N34 treated samples, three peptides were significantly changed upon treatment with 250 ng ELV-N34 and used for dose-response curve calculation. **b,** XIC plots show response of peptide SYDYLVIGGGSGGLASAR.

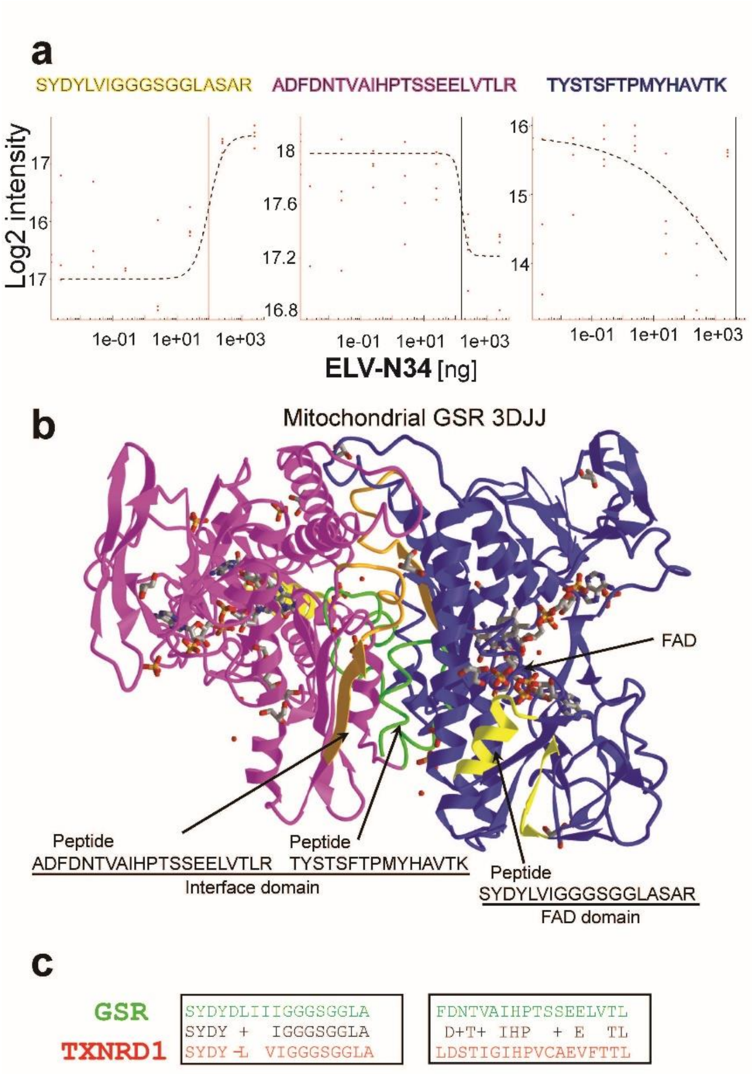

Fig. S2. Analysis of Target Candidate Protein Glutathione reductase, mitochondrial (GSR; P00390). GSR displayed three peptides differentially proteolyzed in contact with ELV-N34. **a,** The plots show the peptide quantity vs. concentration of ELV-N34. The dotted line was determined by non-linear fit regression to obtain ED50 (vertical line). **b,** Structure shown is based on the X-ray chromatographic structure of human glutathione reductase (3DJJ), showing the 3D position of the peptides. **c,** BlastP (NCBI) alignment of GSR peptides against TXNRD1 protein sequence.

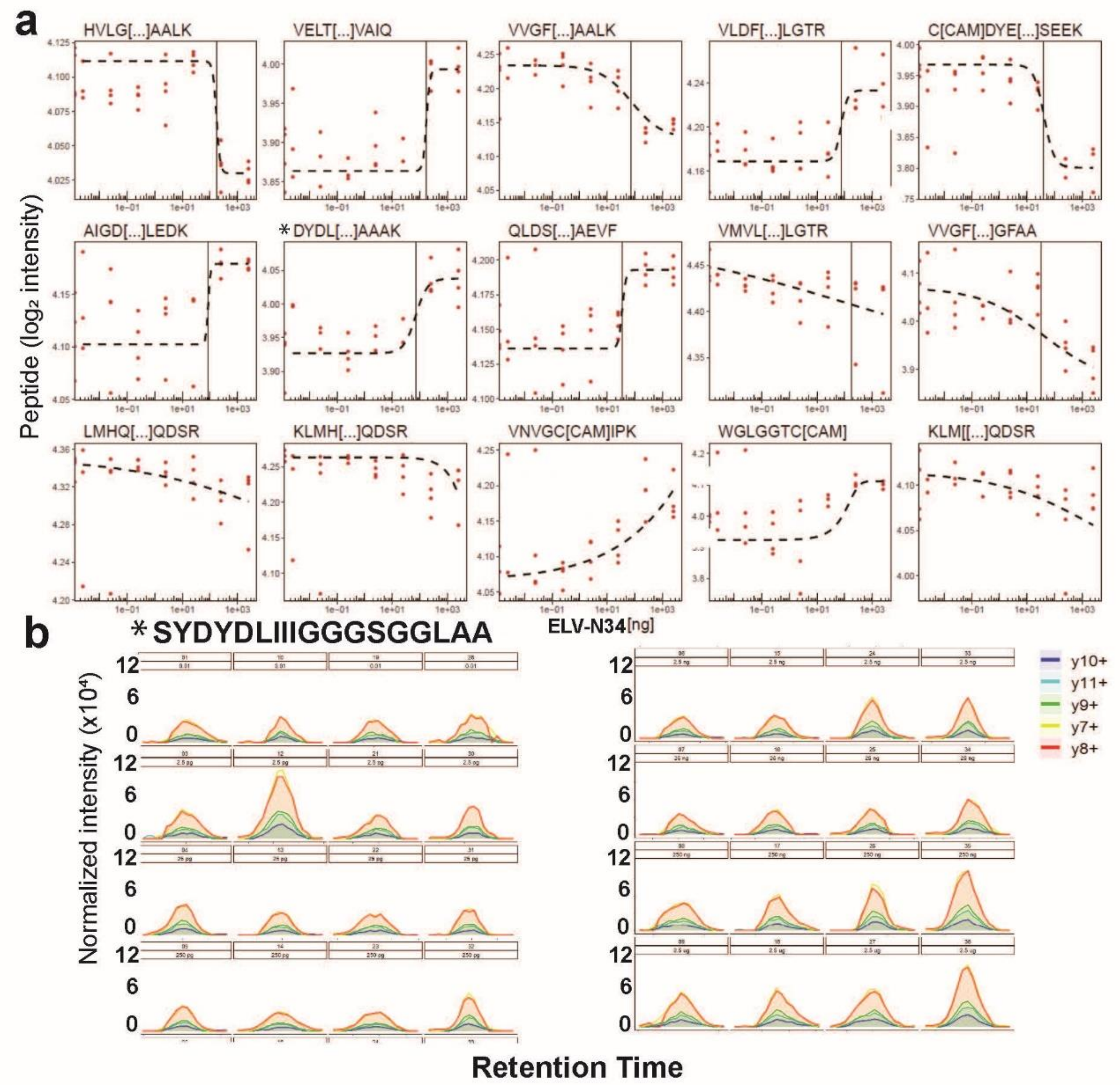

Fig. S3. Glutathione reductase (GSR; P00390). a, Plot shows changes in the top 15 quantified GSR peptides in DMSO and ELV-N34-treated samples. In the Y-axis, the change is represented by the normalized log2 intensity, and the X-axis is the amount of ELV-N34 added in ng per reaction tested. Three peptides were significantly changed upon treatment with 250 ng ELV-N34 and used for dose-response curve calculation. **b,** Extracted ion chromatogram plots show the response of peptide SYDYDLIIIGGGSGGLAA (blue, green, and red lines show three). With an asterisk, we marked the peptide in **a** and **b**.

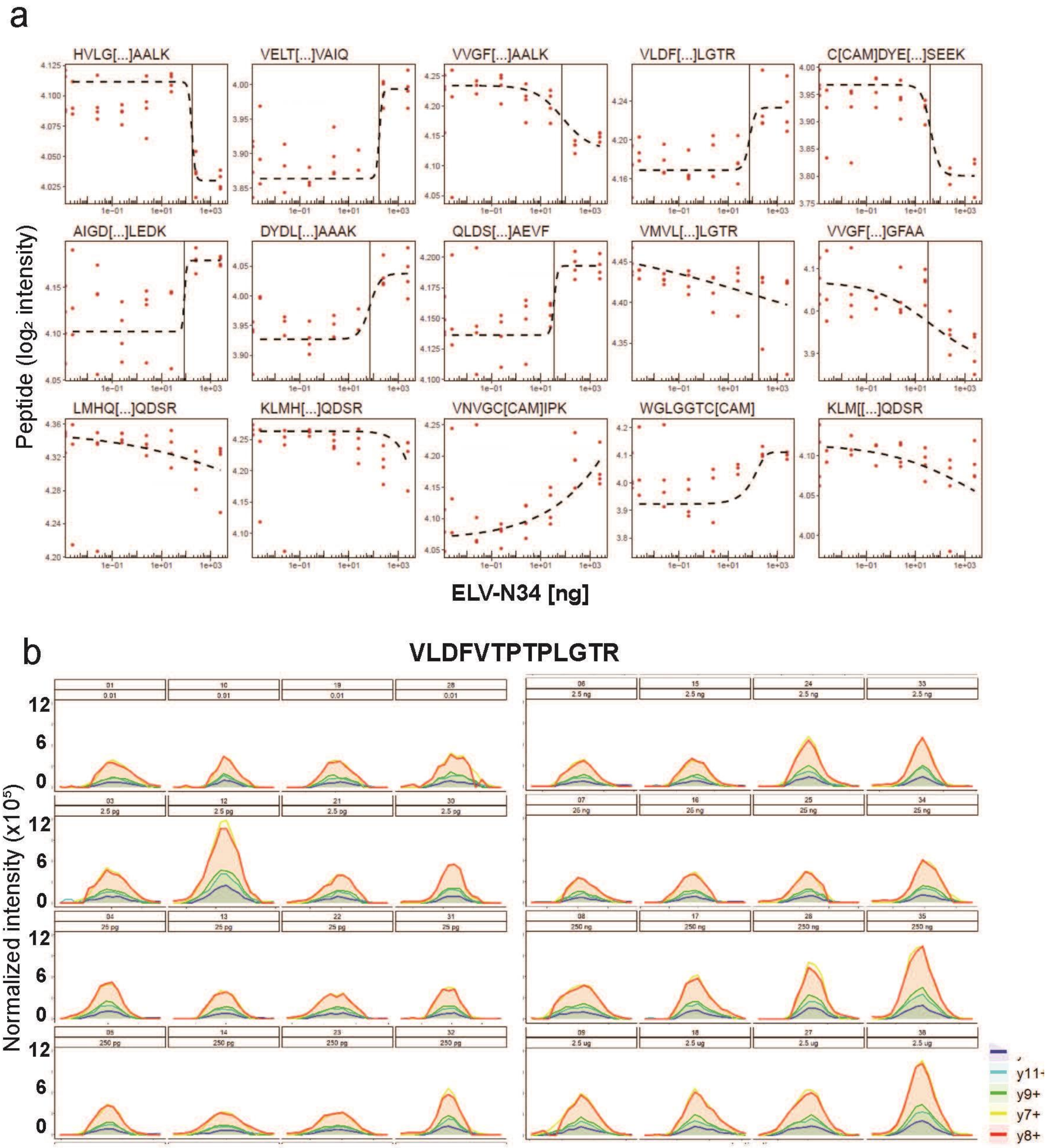

Fig. S4. Thioredoxin reductase 1, cytoplasmic (TXNRD1; Q16881). a, The plots show the fold change of the top 15 quantified TXNRD1 peptides in DMSO and ELV-N34 treated samples. Over 15 peptides were significantly changed upon treatment with 250 ng ELV-N34 and used for dose-response curve calculation. **b,** XIC plots show response of peptide VLDFVTPTPLGTR.

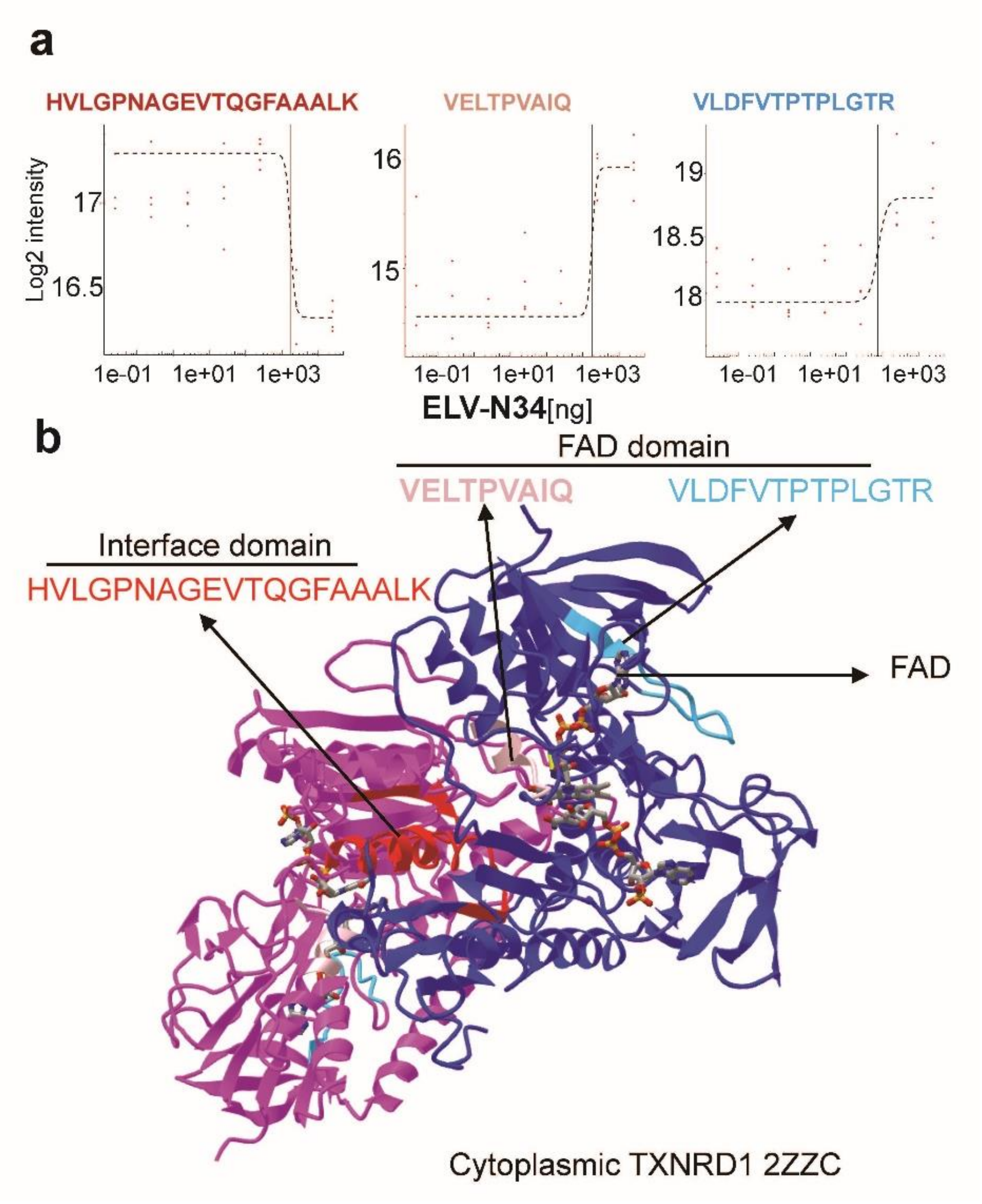

Fig. S5. Thioredoxin reductase, cytoplasmic (TXNRD1; Q16881). a, Plot shows the concentration curves for three of the 15 top quantified TXNRD1 peptides comparing DMSO and ELV-N34 treated samples. **b,** Highlighted peptides over the 3D ribbon model based on the X-ray crystallography (ID: 2ZZC).

1 MGCAEGKAVA AAAPTELQTK GKNGDGRRRS AKDHHPGKTL PENPAGFTST ATADSRALLQ

61 AYIDGHSVVI FSRSTCTRCT EVKKLFKSLC VPYFVLELDQ TEDGRALEGT LSELAAETDL
AAETDL*

AAETDL*

121 PVVFVKQRKI GGHGPTLKAY QEGRLQKLLK MNGPEDLPKS YDYDLIIIGG GSGGLAAAKE
DYDLIIIGG GSGGLAAAK
1
PVVFVK* S YDYDLIIIGG GSGGLAA
1
PVVFVK* S YDYDLIIIGG GSGGLAA E
1

181 AAQYGKKVMV LDFVTPTPLG TRWGLGGTCV NVGCIPKKLM HQAALLGQAL QDSRNYGWKV
VMV LDFVTPTPLG TRWGLGGTCV NVGCIPKKLM HQAALLGQAL QDSR
2 3 4 5
KVMV LDFVTPTPLG TRWGLGGTCV NVGCIPK LM HQAALLGQAL QDSR
2 3 4
AAQYGKK AALLGQAL QDSR
2 3
KVMV LDFVTPTPLG TR WGLGGTCV NVGCIPK
1 2

241 EETVKHDWDR MIEAVQNHIG SLNWGYRVAL REKKVVYENA YGQFIGPHRI KATNNKGKEK

A YGQFIGPHR
5

301 IYSAERFLIA TGERPRYLGI PGDKEYCISS DDLFSLPYCP GKTLVVGASY VALECAGFLA

RFLIA TGERPR
4

361 GIGLDVTVMV RSILLRGFDQ DMANKIGEHM EEHGIKFIRQ FVPIKVEQIE AGTPGRLRVV

IGEHM EEHGIK
6
IGEHM EEHGIK
3

421 AQSTNSEEII EGEYNTVMLA IGRDACTRKI GLETVGVKIN EKTGKIPVTD EEQTNVPYIY

481 AIGDILEDKV ELTPVAIQAG RLLAQRLYAG STVKCDYENV PTTVFTPLEY GACGLSEEKA
AIGDILEDKV ELTPVAIQ CDYENV PTTVFTPLEY GACGLSEEK
6 7 8
AIGDILEDKV ELTPVAIQAG R CDYENV PTTVFTPLEY GACGLSEEK
7 8
AIGDILEDKV ELTPVAIQAG R RLYAG STVKCDYENV PTTVFTPLEY GACGLSEEK
5 6 7

541 VEKFGEENIE VYHSYFWPLE WTIPSRDNNK CYAKIICNTK DNERVVGFHV LGPNAGEVTQ
FGEENIE VYHSYFWPLE WTIPSR
8 9
VVGFHV LGPNAGEVTQ
9

HV LGPNAGEVTQ
4

601 GFAAALKCGL TKKQLDSTIG IHPVCAEVFT TLSVTKRSGA SILQAGCUGG
GFAAALK QLDSTIG IHPVCAEVF
9 10
KQLDSTIG IHPVCAEVF
9
GFAAALK
4

Fig. S6. Aligned fragments fall in common places for most of the peptides identified. Peptides obtained from the LiP study performed with ABC cells undergoing UOS (Fig. 1 and Table S7, green), Erastin (Fig. 2 and Table S9, blue), UOS (Fig. 2 and Table S8, red), and ELV-N34; UOS + NPD1 (purple).

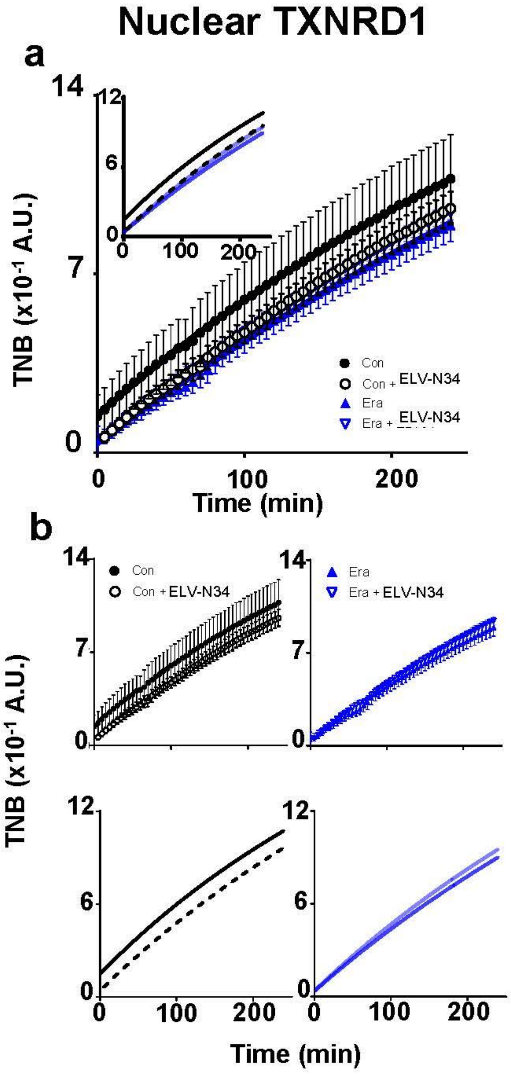

Fig. S7. Curves of Product vs. Time of the nuclear fraction. a-b, 200 nM ELV-N34 was added to the ABC cells exposed to 10 μM Erastin for 2 hours or naïve. The activity of TXNRD1 was recorded on the nuclear fraction obtained by ultracentrifugation as Product (absorbance at 412 nm) vs. Time (min). Reductase activity from sources other than TXNRD1 was ruled out using a specific inhibitor. **a,** Merge curves of the four conditions. **b,** Mean of three observations and the bars represent the standard error of the mean for Control and Control + ELV-N34 (**b**) and UOS and UOS + ELV-N34 The non-linear regression curves obtained for the data points in the lower panels using GraphPad 10.0.

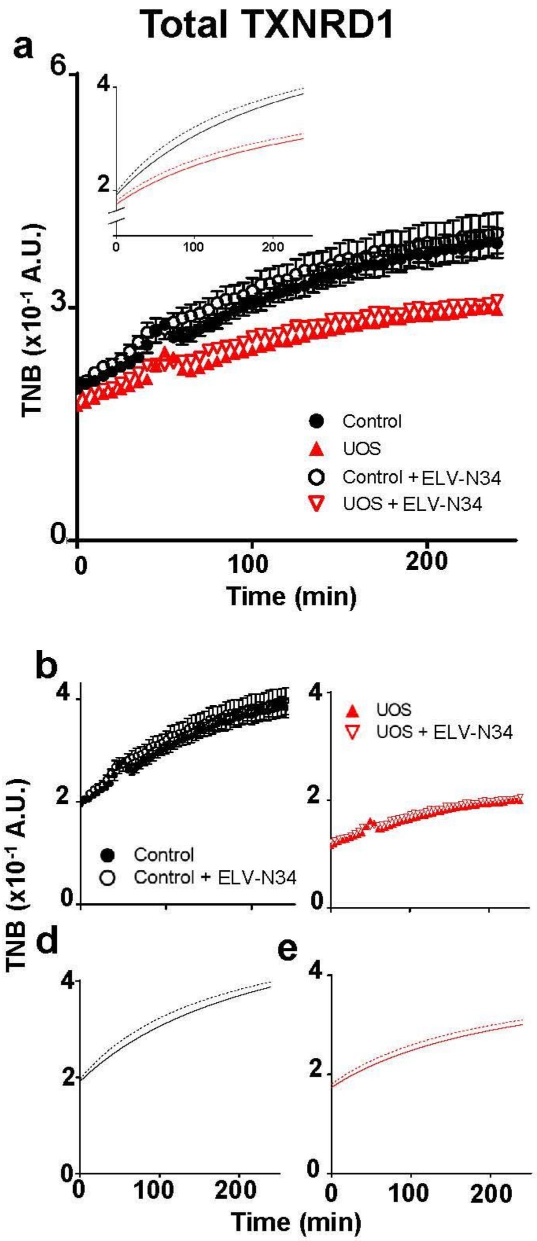

Fig. S8. Curves of Product vs. Time of the total extract. a-b, 200 nM ELV-N34 was added to the ABC cells exposed to 1600 µM H_2O_2 and 10ng/ml TNFα for 2 hours or naïve. The activity of TXNRD1 was recorded on the total extract of the cells exposed to the mentioned treatments as Product (absorbance at 412 nm) vs. Time (min). Reductase activity from sources other than TXNRD1 was ruled out using a specific inhibitor. **a,** Merge curves of the four conditions. **b,** Mean of three observations and the bars represent the standard error of the mean for Control and Control + ELV-N34 (**b**) and UOS and UOS + ELV-N34 The non-linear regression curves obtained for the data points in the lower panels using GraphPad 10.0.

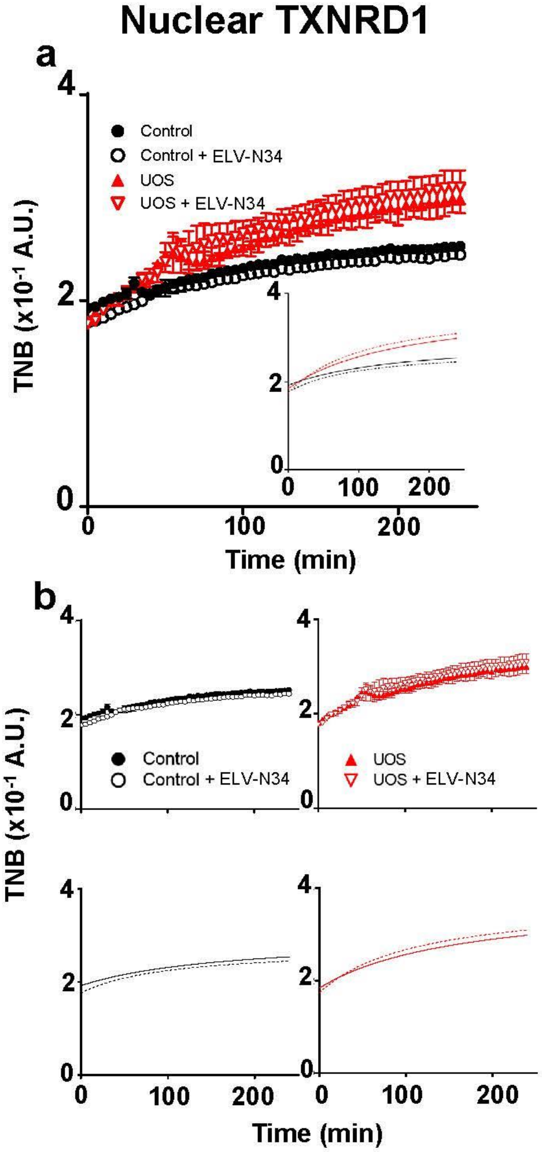

Fig. S9. Curves of Product vs. Time of the nuclear fraction. a-b, 200 nM ELV-N34 was added to the ABC cells exposed to 1600 µM H_2O_2 and 10ng/ml TNFα for 2 hours or naïve. The activity of TXNRD1 was recorded on the nuclear fraction obtained by ultracentrifugation as Product (absorbance at 412 nm) vs. Time (min). Reductase activity from sources other than TXNRD1 was ruled out using a specific inhibitor. **a,** Merge curves of the four conditions. **b,** Mean of three observations and the bars represent the standard error of the mean for Control and Control + ELV-N34 (**b**) and UOS and UOS + ELV-N34 The non-linear regression curves obtained for the data points in the lower panels using GraphPad 10.0.

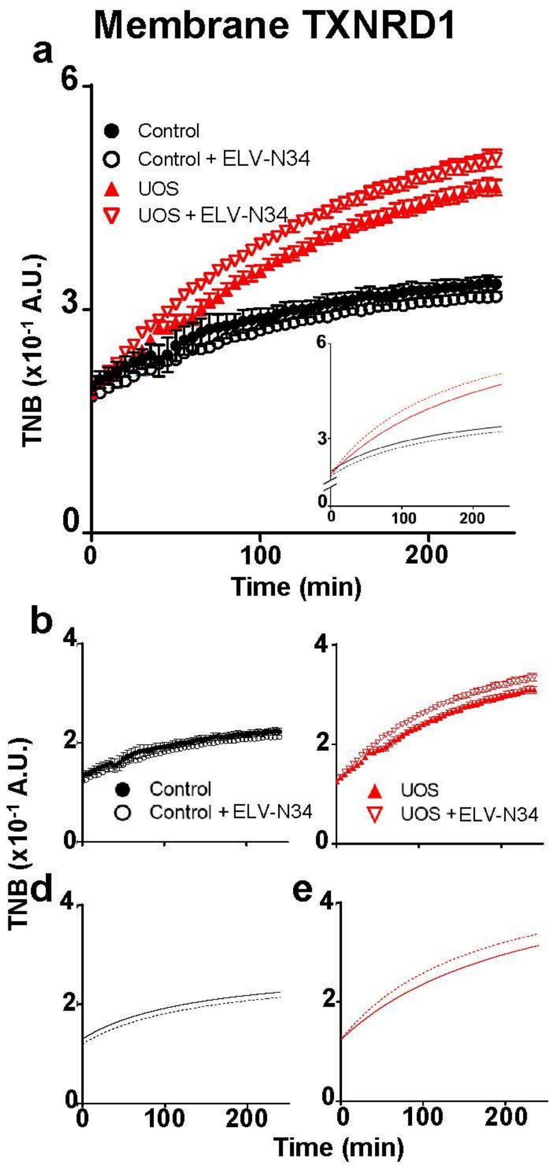

Fig. S10. Curves of Product vs. Time of the membrane fraction. a-b, 200 nM ELV-N34 was added to the ABC cells exposed to 1600 μM H_2O_2 and 10ng/ml TNFα for 2 hours or naïve. The activity of TXNRD1 was recorded on the membrane fraction obtained by ultracentrifugation as Product (absorbance at 412 nm) vs. Time (min). Reductase activity from sources other than TXNRD1 was ruled out using a specific inhibitor. **a,** Merge curves of the four conditions. **b,** Mean of three observations and the bars represent the standard error of the mean for Control and Control + ELV-N34 (**b**) and UOS and UOS + ELV-N34. The non-linear regression curves obtained for the data points in the lower panels using GraphPad 10.0.

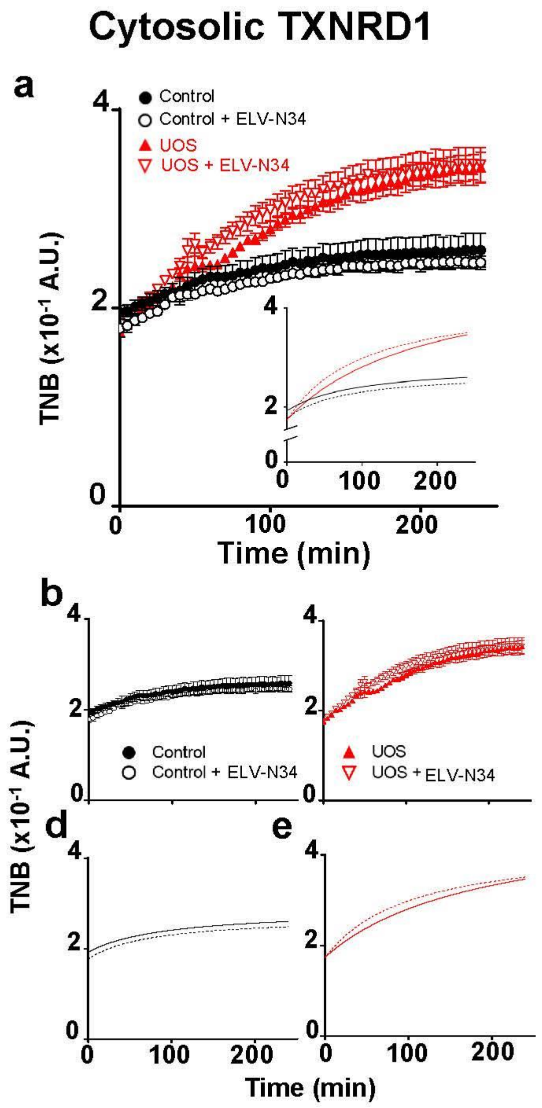

Fig. S11. Curves of Product vs. Time of the cytosolic fraction. a-b, 200 nM ELV-N34 was added to the ABC cells exposed to 1600 µM H_2O_2 and 10ng/ml TNFα for 2 hours or naïve. The activity of TXNRD1 was recorded on the cytosolic fraction obtained by ultracentrifugation as Product (absorbance at 412 nm) vs. Time (min). Reductase activity from sources other than TXNRD1 was ruled out using a specific inhibitor. **a,** Merge curves of the four conditions. **b,** Mean of three observations and the bars represent the standard error of the mean for Control and Control + ELV-N34 (**b**) and UOS and UOS + ELV-N34 The non-linear regression curves obtained for the data points in the lower panels using GraphPad 10.0.

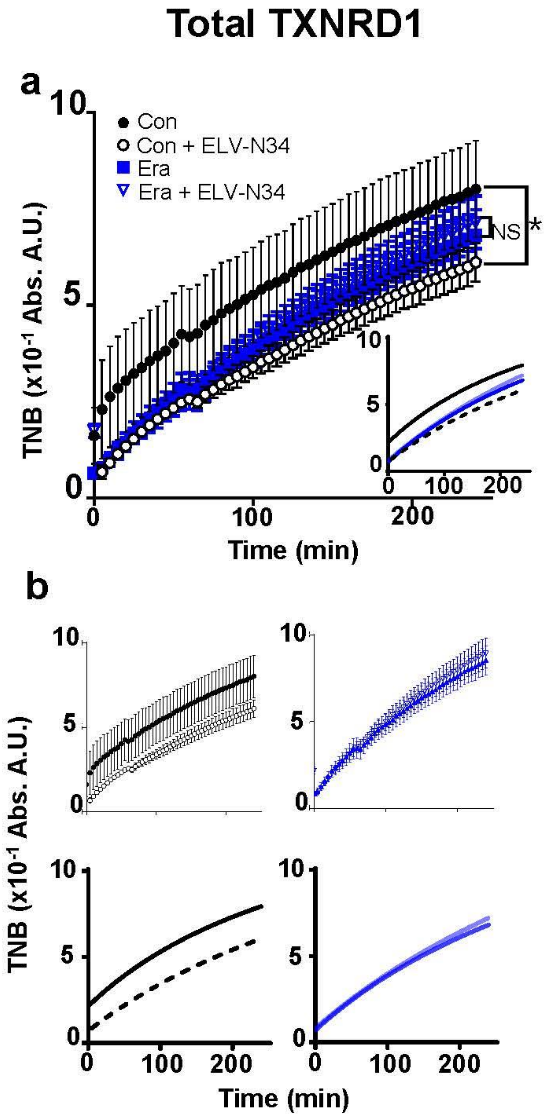

Fig. S12. Curves of Product vs. Time of the total extract. a-b, 200 nM ELV-N34 was added to the ABC cells exposed to 10µM Erastin for 24 hours or naïve. The activity of TXNRD1 was recorded on the total extract as Product (absorbance at 412 nm) vs. Time (min). Reductase activity from sources other than TXNRD1 was ruled out using a specific inhibitor. **a,** Merge curves of the four conditions. **b,** Mean of three observations and the bars represent the standard error of the mean for Control and Control + ELV-N34 (**b**) and UOS and UOS + ELV-N34. The non-linear regression curves obtained for the data points in the lower panels using GraphPad 10.0.

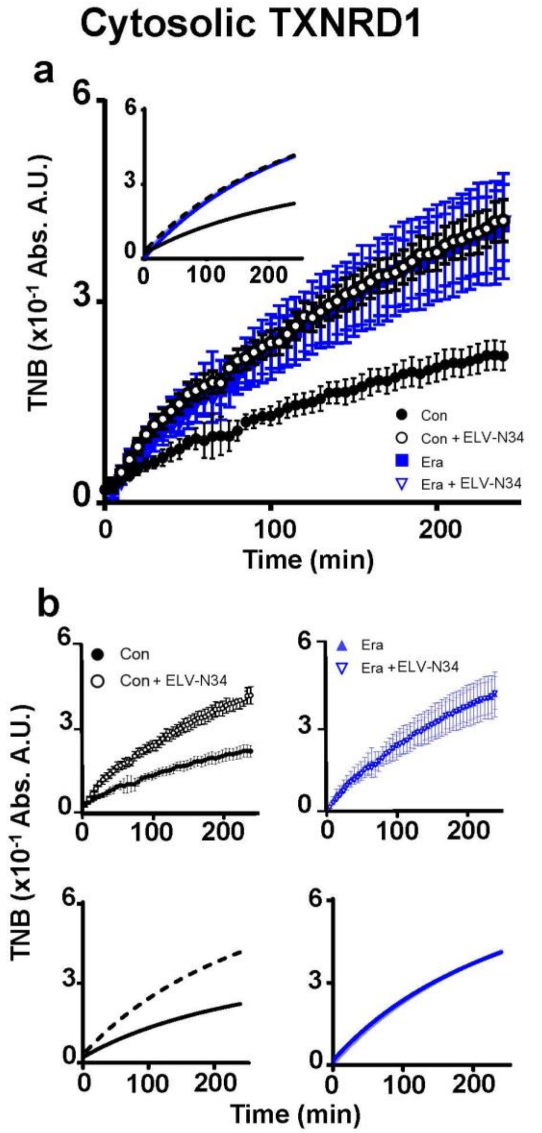

Fig. S13. Curves of Product vs. Time of the cytosolic fraction. a-b, 200 nM ELV-N34 was added to the ABC cells exposed to 10 µM Erastin for 24 hours or naïve. The activity of TXNRD1 was recorded on the cytosolic fraction obtained by ultracentrifugation as Product (absorbance at 412 nm) vs. Time (min). Reductase activity from sources other than TXNRD1 was ruled out using a specific inhibitor. **a,** Merge curves of the four conditions. **b,** Mean of three observations and the bars represent the standard error of the mean for Control and Control + ELV-N34 (**b**) and UOS and UOS + ELV-N34 The non-linear regression curves obtained for the data points in the lower panels using GraphPad 10.0.

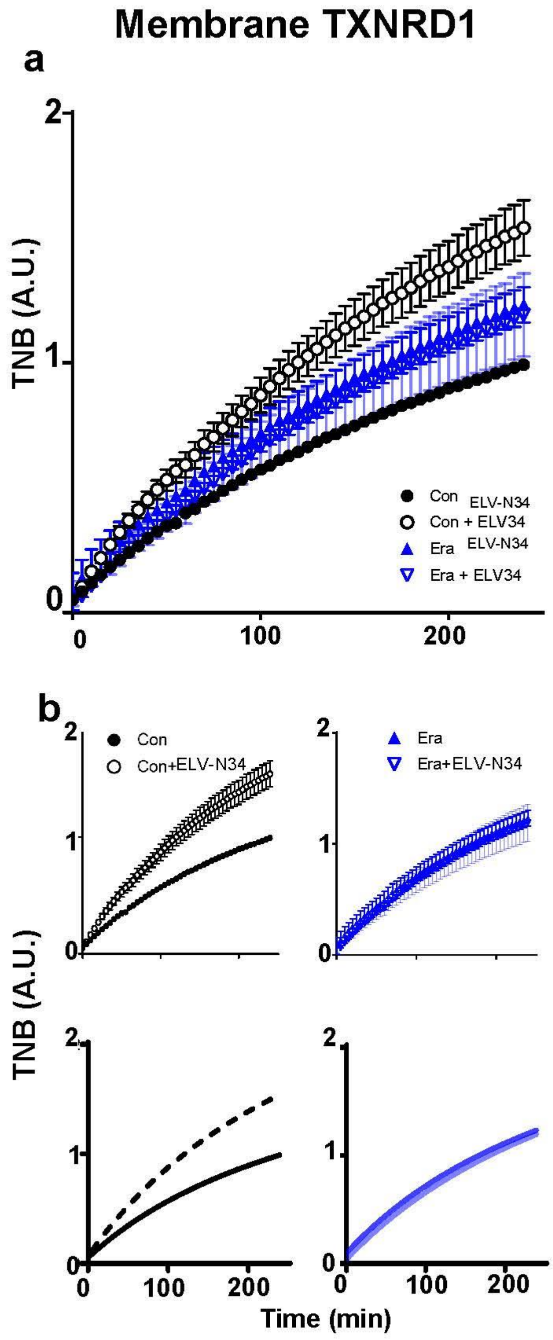

Fig. S14. Curves of Product vs. Time of the membrane fraction. a-b, 200 nM ELV-N34 was added to the ABC cells exposed to 10 µM Erastin for 2 hours or naïve. The activity of TXNRD1 was recorded on the membrane fraction obtained by ultracentrifugation as Product (absorbance at 412 nm) vs. Time (min). Reductase activity from sources other than TXNRD1 was ruled out using a specific inhibitor. **a,** Merge curves of the four conditions. **b,** Mean of three observations and the bars represent the standard error of the mean for Control and Control + ELV-N34 (**b**) and UOS and UOS + ELV-N34 The non-linear regression curves obtained for the data points in the lower panels using GraphPad 10.0.

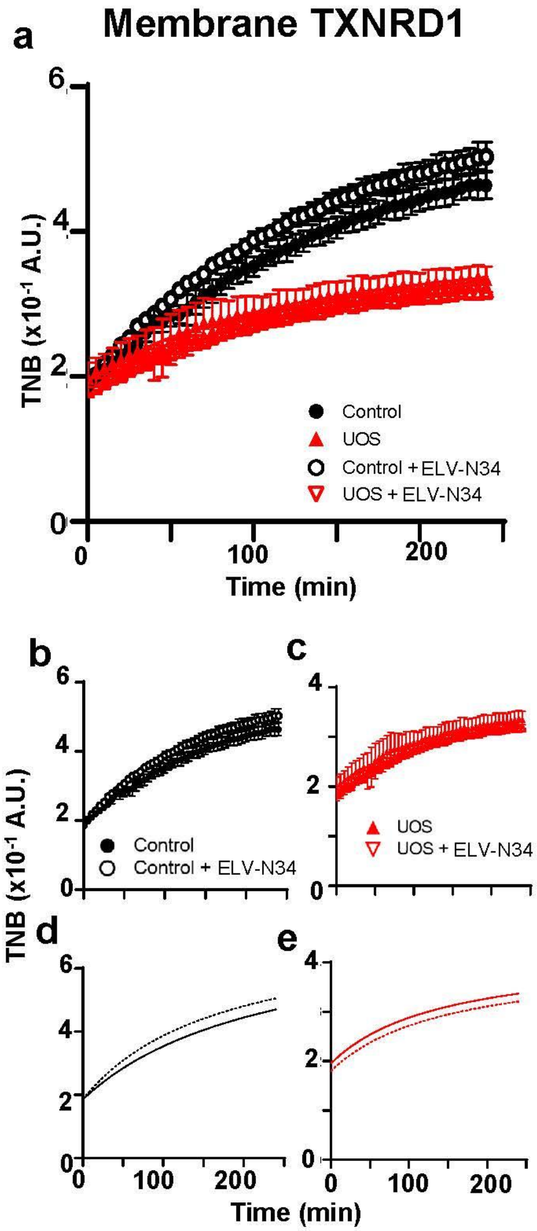

Fig. S15. Addition of ELV-N34 to the membrane fractions. Curves of Product vs. Time were used to construct the plot in Fig. 7A (upper panel). **a-b,** 250 ng of ELV-N34 per 100 ng of membrane fraction protein in 200 µl reaction was added. ABC cells exposed for 6 hours to 1600 µM H_2O_2 and 10ng/ml TNFα or naïve were processed by ultracentrifugation to separate the fractions for the activity of TXNRD1 to be recorded as Product (absorbance at 412 nm) vs. Time (min), and the subtraction resulting from the reaction and the reaction plus inhibitor was plotted using GraphPad 10.0.

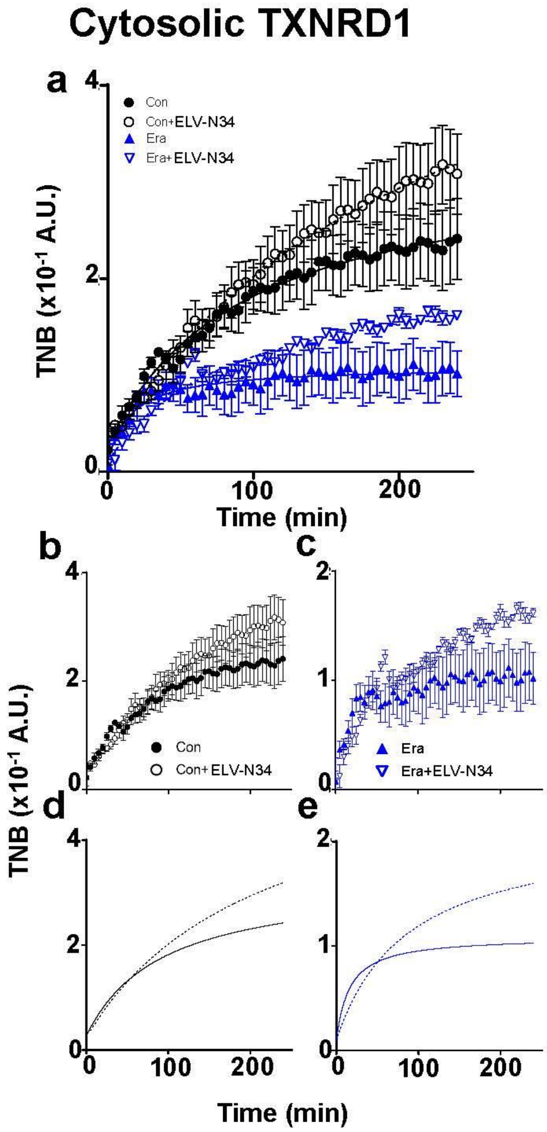

Fig. S16. Addition of ELV-N34 to the cytosolic fractions. Curves of Product vs. Time were used to construct the plot in Fig. 7B (upper panel). **a-b,** 250 ng of ELV-N34 per 100 ng of cytosolic protein in 200 µl reaction was added. ABC cells exposed for 24 hours to Erastin (10µM) or naïve were processed by ultracentrifugation to separate the fractions for the activity of TXNRD1 to be recorded as Product (absorbance at 412 nm) vs. Time (min), and the subtraction resulting from the reaction and the reaction plus inhibitor was plotted using GraphPad 10.0.

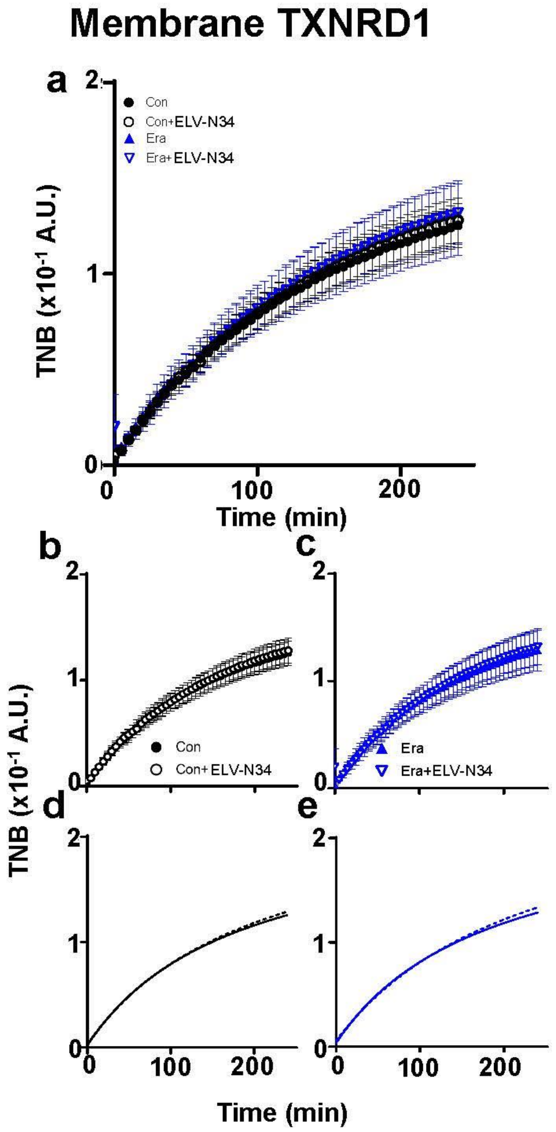

Fig. S17. Addition of ELV-N34 to the membrane fractions. Curves of Product vs. Time were used to construct the plot in Fig. 7B (lower panel). **a-b,** 250 ng of ELV-N34 per 100 ng of membrane fraction protein in 200 µl reaction was added. ABC cells exposed 24 hours to Erastin (10µM) or naïve were processed by ultracentrifugation to separate the fractions for the activity of TXNRD1 to be recorded as Product (absorbance at 412 nm) vs. Time (min), and the subtraction resulting from the reaction and the reaction plus inhibitor was plotted using GraphPad 10.0.

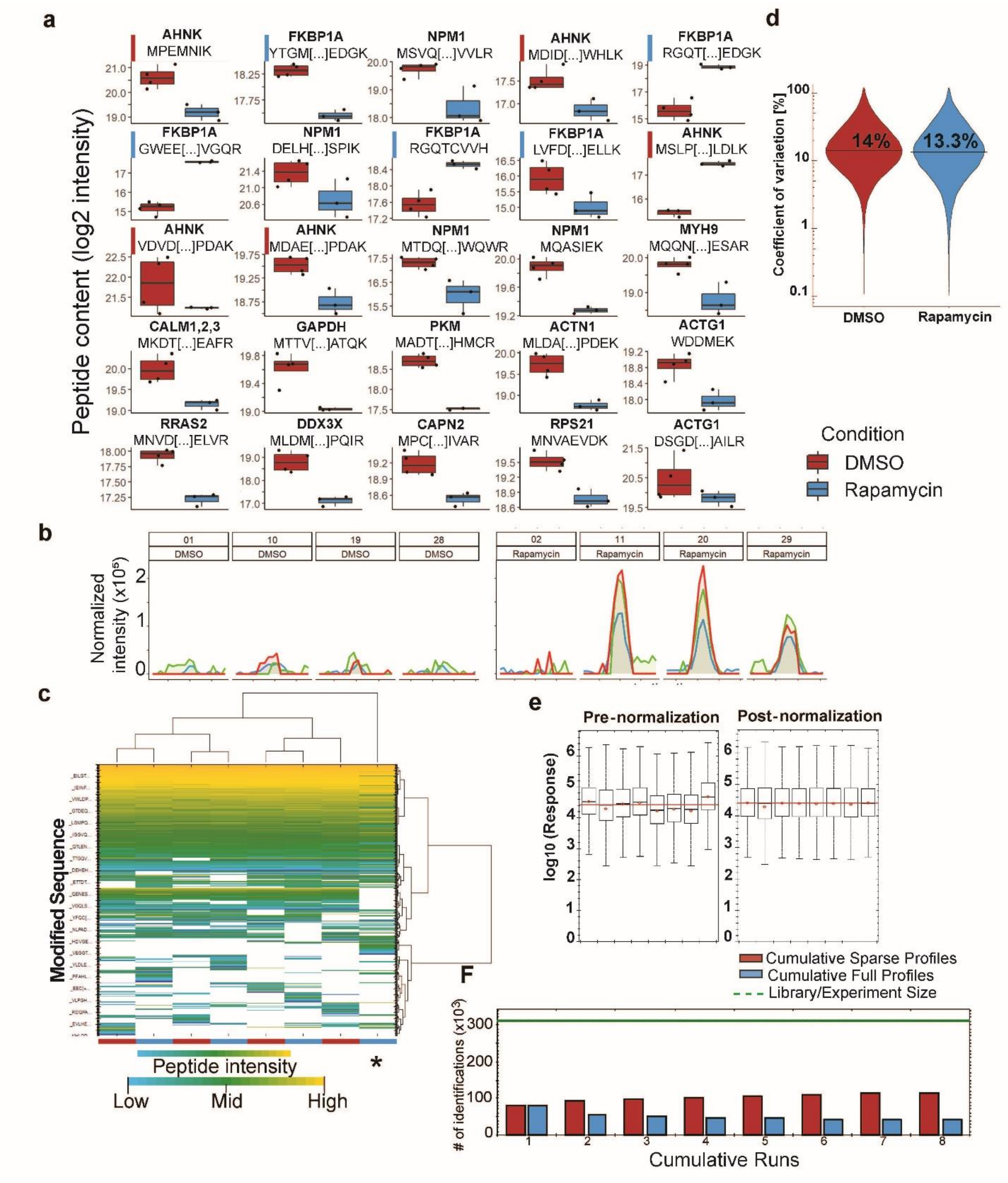

Fig. S18. Specificity control using Rapamycin instead of ELV-N34 for the first set of samples. The already known amount of 1’135 peptides was significantly changed between samples treated with DMSO and Rapamycin.

a, Box plot of the top 25 peptides and corresponding proteins significantly changed between DMSO and Rapamycin sample groups with 5 out of 25 high-ranking responder peptides. The candidate peptides shown were ranked by q-value. Data is grouped by sample groups and shown as box plots where the middle line depicts the average, and the limit of the boxes are the 1st and 3rd quartile. The bars show the standard deviation of the group.

b, The known target of Rapamycin Peptidyl-prolyl cis-trans isomerase FKBP1A (FKBP1A; P62942). FKBP1A is identified, and extracted ion chromatogram plots show the response of peptide RGQTCVVHYTGMLEDGK^{2+}.

c, Hierarchical Clustering (Rapamycin Control), unsupervised clustering of all peptide intensities does not reconstruct sample groups; the Manhattan distance measure using all peptide intensities across all samples was carried out. Clustered data is displayed as a heat map. Sample dendrogram (top) displays no separation according to sample group, indicating that no sample preparation or data acquisition artifact is confounding the analysis. One sample (*) was excluded from analysis due to excessive variance.

d, Technical variation: Median technical variation was between 13.3 and 14.0 % within sample groups. Violin plot shows distribution of coefficients of variance (CV) for peptide quantification, grouped by sample group.

e, Normalization: Minimal normalization was performed across all runs and was successful in reducing experimental variation.

f, Number of precursors quantified across all runs. Data completeness is referred to as precursors quantified by HRM in the samples provided. Cumulative sparse profiles account for all precursors in the data set quantified at least once across the samples with high confidence (sparse data set). Cumulative full profiles account for all precursors in the data set quantified in each single sample with high confidence (complete data set). Size of the spectral library is shown as a dotted line.

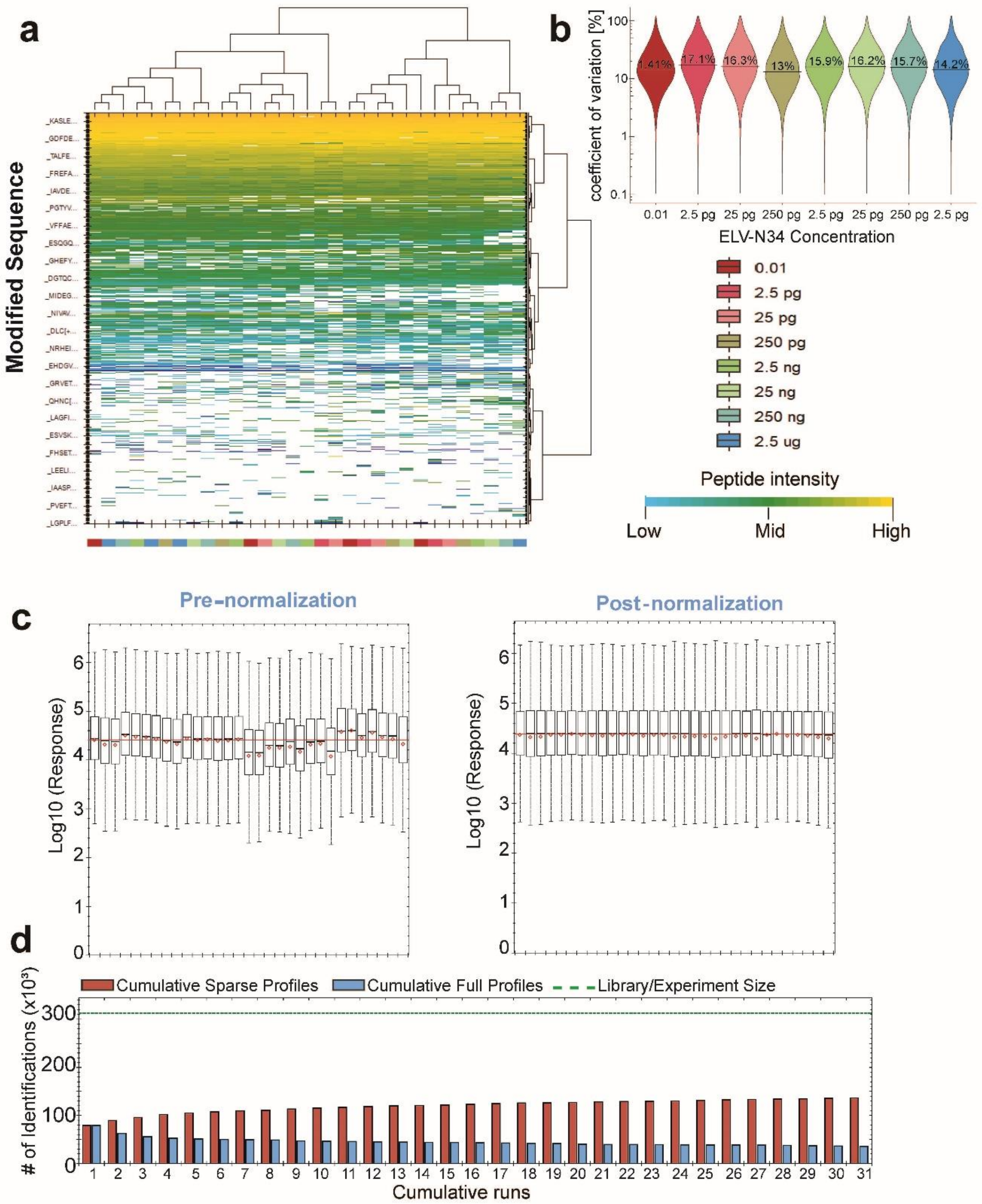

a
b
c
d
Modified Sequence
_KASLE...
_GDFDE...
_TALFE...
_FREFA...
_IAVDE...
_PGTYV...
_VFFAE...
_ESQGQ...
_GHEFY...
_DGTQC...
_MIDEG...
_NIVAV...
_DLC[+...
_NRHEI...
_EHDGV...
_GRVET...
_QHNC[...
_LAGFI...
_ESVSK...
_FHSET...
_LEELI...
_IAASP...
_PVEFT...
_LGPLF...
coefficient of variation [%]
100
10
1
0.1
1.41%
17.1%
16.3%
13%
15.9%
16.2%
15.7%
14.2%
0.01
2.5 pg
25 pg
250 pg
2.5 pg
25 pg
250 pg
2.5 pg
ELV-N34 Concentration
0.01
2.5 pg
25 pg
250 pg
2.5 ng
25 ng
250 ng
2.5 ug
Peptide intensity
Low
Mid
High
Pre-normalization
Post-normalization
Log10 (Response)
0
1
2
3
4
5
6
Cumulative Sparse Profiles
Cumulative Full Profiles
Library/Experiment Size
# of Identifications (x10³)
0
100
200
300
1 2 3 4 5 6 7 8 9 10 11 12 13 14 15 16 17 18 19 20 21 22 23 24 25 26 27 28 29 30 31
Cumulative runs

Fig. S19. Quality control of the first set of samples.

a, Unsupervised clustering of 105’185 peptide intensities does not reconstruct sample groups. Hierarchical clustering analysis using the Manhattan distance measure using all peptide intensities across all samples was carried out. Clustered data is displayed as a heat map. Sample dendrogram (top) displays no separation according to sample group, indicating that no sample preparation or data acquisition artifact is confounding the analysis.

b, Technical variation of target deconvolution experiment was assessed as coefficient of variance for peptide quantities between sample groups. Median technical variation was between 13.0 and 17.1 % within sample groups. Violin plot shows the distribution of coefficients of variation (CV) for peptide quantification, grouped by sample group.

c, Minimal normalization was performed across all runs and was successful in reducing experimental variation.

d, Number of precursors quantified across all runs. Data completeness is referred to as precursors quantified by HRM in the samples provided. Cumulative sparse profiles account for all precursors in the data set quantified at least once across the samples with high confidence (sparse data set). Cumulative full profiles account for all precursors in the data set quantified in each single sample with high confidence (complete data set). Size of the spectral library is shown as a dotted line.

A

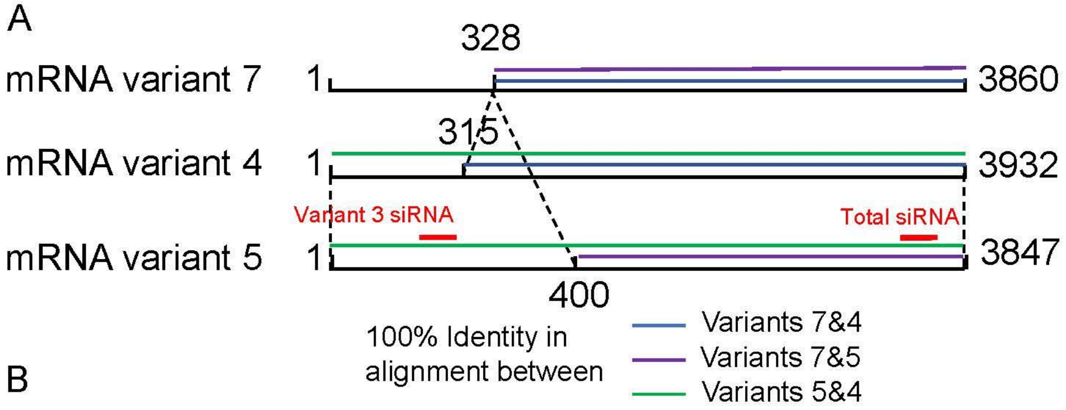

B

```
Isoform 5    1    MGCAEGKAVAAAAPTELQTKGKNGDGRRRSAKDHHPGKTLPENPAGFTSTATADSRALLQ
Isoform 2    1    MSC---------------------------------------------------------
Isoform 3    1    M-----------------------------------------------------------

Isoform 5   61    AYIDGHSVVIFSRSTCTRCTEVKKLFKSLCVPYFVLELDQTEDGRALEGTLSELAAETDL
Isoform 2    4    ----------------------------------------EDGRALEGTLSELAAETDL
Isoform 3    2    ----------------------------------------EDGRALEGTLSELAAETDL
                                                          └→ 100% Identity

Isoform 5  642    ILQAGCUG   649  }
Isoform 2  544    ILQAGCUG   551  } End of the proteins
Isoform 3  542    ILQAGCUG   549  }
```

C

Products on target templates from PrimerBLAST

```
>NM_003330.4 Homo sapiens thioredoxin reductase 1 (TXNRD1),
transcript variant 4, mRNA

product length = 25
Forward primer  1    CCGGTGACACAAAGCTTCAGCATGT  25
Template        285  .....C...................  309

Reverse primer  1    ACATGCTGAAGCTTTGTGTGACCGG  25
Template        309  .........................  285


>NM_001261445.2 Homo sapiens thioredoxin reductase 1 (TXNRD1),
transcript variant 5, mRNA

product length = 25
Forward primer  1    CCGGTGACACAAAGCTTCAGCATGT  25
Template        285  .....C...................  309

Reverse primer  1    ACATGCTGAAGCTTTGTGTGACCGG  25
Template        309  .........................  285
```

Fig. S20. Alignment of siRNA used to silence Isoforms 2/3 and total TXNRD1. a, Transcript variants 7, 5, and 4 that give rise to the isoforms 5, 3, and 2, respectively, were aligned against each other to show areas of different sequences used to design the specific siRNAs. **b,** Alignment of the protein sequence of the isoforms 5, 3, and 2. **c,** Alignment of the siRNA to the TXNRD1 transcript 5 and 4 to show specific binding and thus silencing of only these two transcripts. More information on transcripts and corresponding isoforms in Table S13.

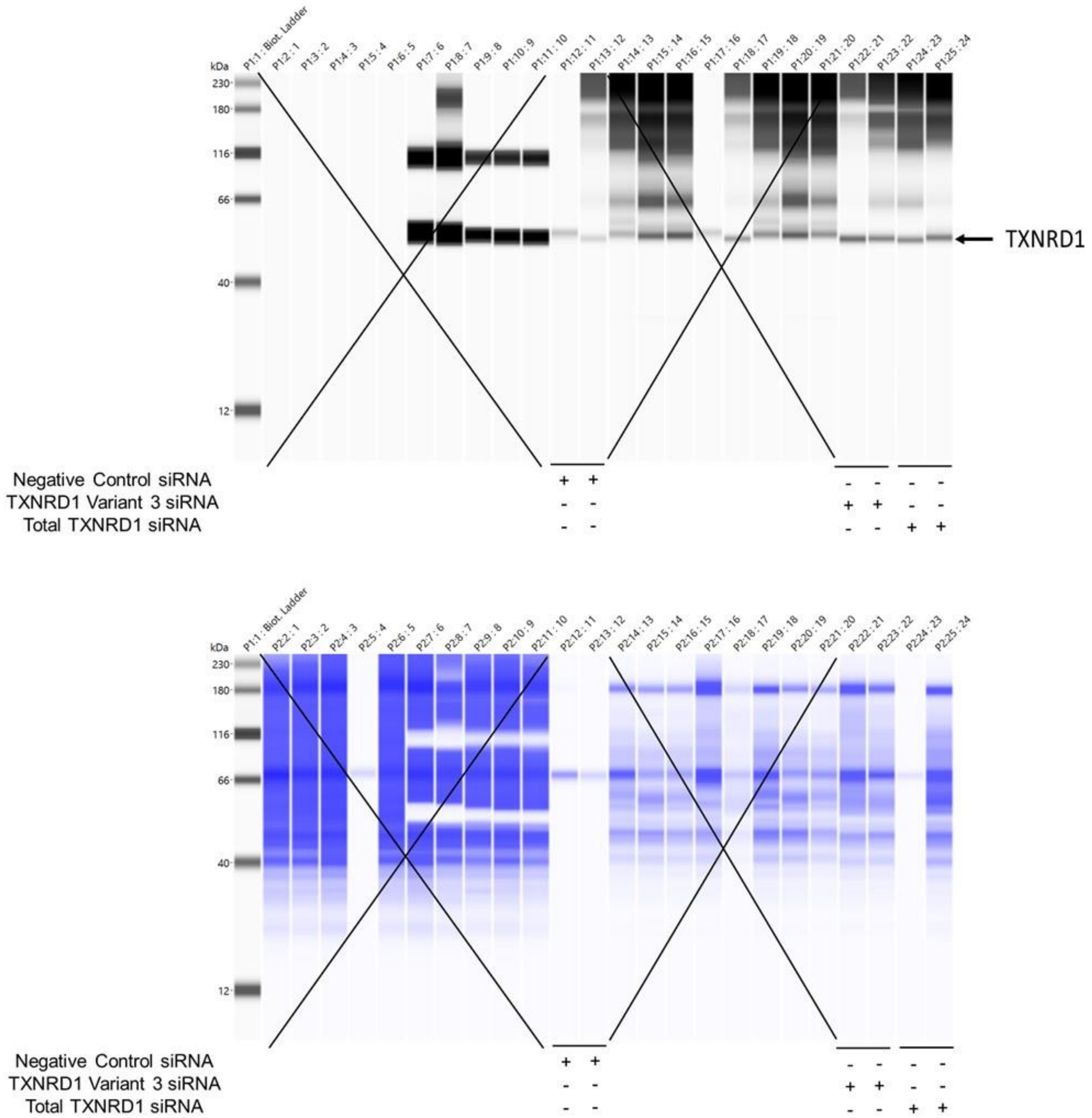

Fig. S21. Whole capillary blots with markers used in Fig. 6 to assess silencing of TXNRD1 Isoforms 2/3 and total TXNRD1. The upper blot shows TXNRD1 (arrow) running close to the markers at its predicted size of 55KDa. Lower blot is the total protein staining used to standardize the bands. The lysates were run using Jess capillary western blot Chemiluminescence staining, and the position of the bands and quantification were performed using ProteinSimple protocols and software for Jess western blot analysis.
