# Supplementary figures and images for: Elovanoid-N34 modulates TXNRD1 key in protection against oxidative stress-related diseases
Source: Cell Death Dis. 2023 Dec 13;14(12):819. doi: 10.1038/s41419-023-06334-6 (PMC10716158; doi:10.1038/s41419-023-06334-6)

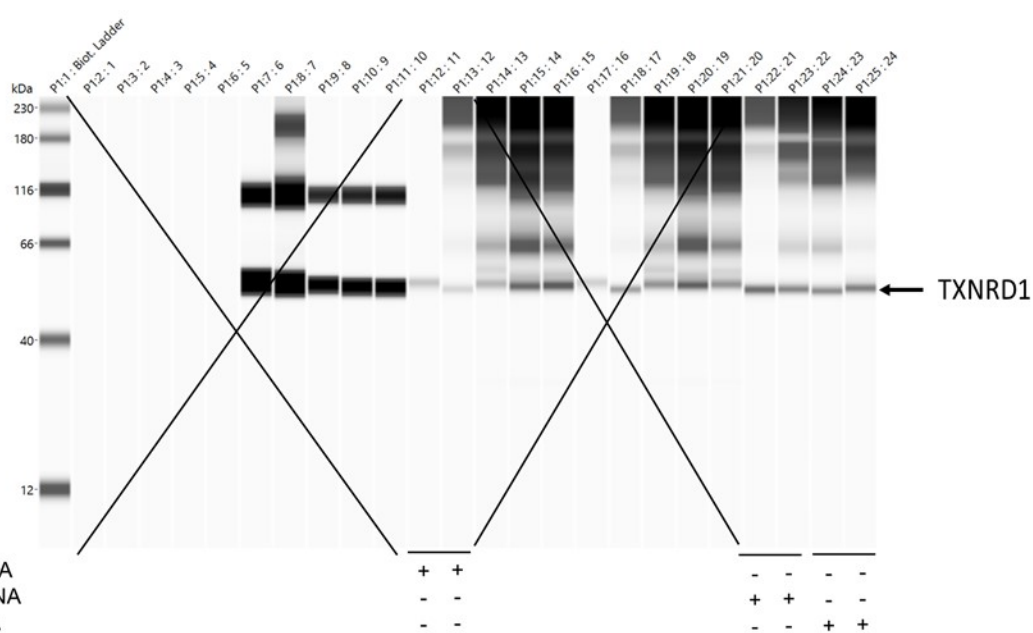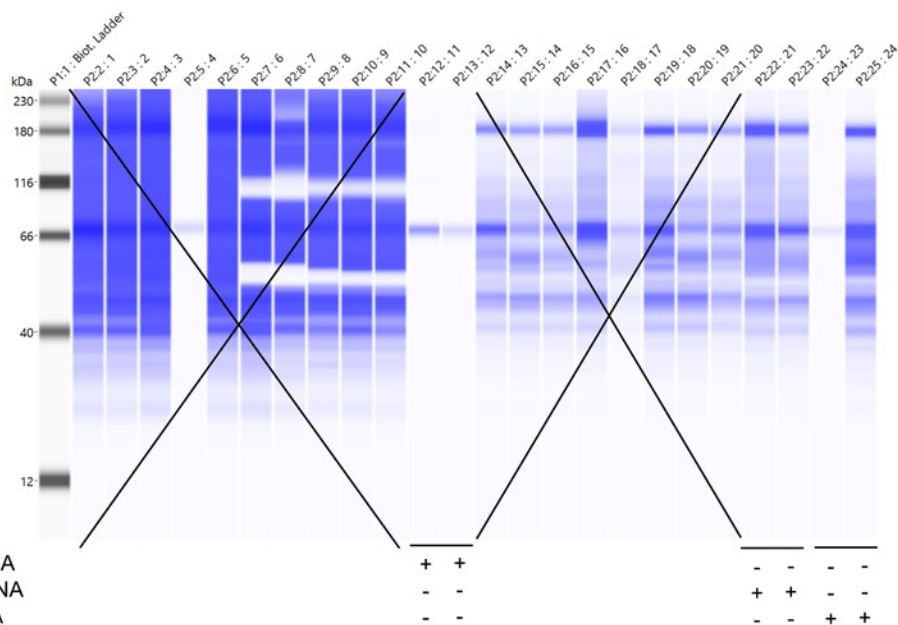

Supplement: Supplementary file 16 — Original Data File [file 41419_2023_6334_MOESM16_ESM.pdf]
